# Supplementary material for: A deep siamese neural network improves metagenome-assembled genomes in microbiome datasets across different environments
Source: Nat Commun. 2022 Apr 28;13:2326. doi: 10.1038/s41467-022-29843-y (PMC9051138; doi:10.1038/s41467-022-29843-y)
Supplement: Supplementary file 1 — Supplementary Information [file 41467_2022_29843_MOESM1_ESM.pdf]

Supplementary Information

A deep siamese neural network improves metagenome-assembled genomes in microbiome datasets across different environments.

Shaojun Pan [1,2], **Chengkai Zhu** [1,2,3], **Xing-Ming Zhao** [1,2,4,5,*], **and Luis Pedro Coelho** [1,2,*]

[1]Institute of Science and Technology for Brain-Inspired Intelligence, Fudan University, Shanghai, China.

[2]Key Laboratory of Computational Neuroscience and Brain-Inspired Intelligence, Ministry of Education, Ministry of Education, Shanghai, China.

[3]School of Life Sciences, Fudan University, Shanghai, China.

[4]MOE Frontiers Center for Brain Science, Fudan University, Shanghai, China.

[5]Zhangjiang Fudan International Innovation Center, Shanghai, China.

[*]to whom correspondence should be addressed: xmzhao@fudan.edu.cn and luispedro@big-data-biology.org

1 Supplementary Note

1.1 Noise and bias of constraint generation from taxonomic contig annotations

Following taxonomic annotation of contigs, cannot-link constraints between contigs were automatically extracted (see Methods). We also attempted to extract must-link constraints by defining pairs of contigs with the same annotations at the species level (with scores both above 0.95) as must-link pairs. In the CAMI simulated data, it is possible to evaluate the accuracy and coverage of these annotations. We observed that cannot-link constraints had very high accuracy and, thus, they are used for training (see Supplementary Table 2). For must-link constraints, however, there are two issues. First, for most situations, the accuracy of must-link constraints was low, which would lead to noise in the model training. Second, the must-link constraints only covered a small part of the genomes in the environment and it would lead to bias in the learning of the model. Owing to the noise and bias of the must-link constraints obtained from taxonomic annotations, we chose to not use them and instead to generate must-link constraints by breaking up long contigs.

1.2 Robustness of results to changes in the number of must-link and cannot-link constraints

To show the effect of different numbers of must-link and cannot-link constraints for the training, we used different minimum size threshold (> 1,000bp, > 4,000bp, > 10,000bp) for generating must-link constraints and different numbers (10,000bp, 500,000bp, 4,000,000bp, 10,000,000bp) of cannot-link constraints. We compared the number of high-quality bins obtained from the CAMI I datasets in these situations (see Supplementary Fig. 20).

SemiBin can be robust to most situations in the low, medium and high complexity datasets, except when the number of cannot-link constraints and the minimum size threshold for must-link constraints is too small. The reason is that breaking up short contigs will lead to noise for the training (so we set the minimum threshold to 4,000bp as default) and the very small number of cannot-link constraints cannot provide enough information for the contrastive training[1].

1.3 Influence of changes in the max_edges parameter on results

The max_edges parameter controls the number of edges of each node (each contig) in the graph that will be considered during the clustering step. To show the influence of changing the max_edges parameter to the binning results, we benchmarked different max_edges parameter values (200, 500, 1000) on the CAMI I (see Supplementary Fig. 21), CAMI II and real datasets (see Supplementary Fig. 22). In the simulated and ocean datasets, the final binning results were robust to different max_edges values (slight influence in the ocean datasets). In the human and dog gut dataset, the results were slightly worse when max_edges was larger. Considering these results and computation costs, we set the default value of the max_edges parameter to 200.

We also compared the parameter max_edges in Metabat2 and SemiBin on CAMI I datasets. Both SemiBin and Metabat2

were robust to the setting of max_edges. Nonetheless, SemiBin could reconstruct on average 26.0%, 11.9% and 36.4% more high-quality bins (see Supplementary Fig. 21) than Metabat2.

Compared to NoSemi with different max_edges parameters, SemiBin was more robust as the results of NoSemi deteriorated with the increasing number of edges (see Supplementary Fig. 3), further demonstrating the value of semi-supervised learning.

1.4 Evaluation of SemiBin with different clustering methods

There are two clustering steps in the SemiBin pipeline. First, SemiBin converts the embeddings to a graph and uses the Infomap community detection algorithm which was already used in another binning tool (Bin3C[2]). Then, SemiBin uses weighted *k*-means to recluster bins whose number of single-copy genes is greater than one.

For the community detection step, we tested a few alternative algorithms, namely Label propagation[3], Leiden[4] and Louvain[5] community detection algorithm (these are the algorithms available in the igraph package[6]). Except for the Leiden algorithm, which does not appear appropriate to this setting, the other methods showed similar results in the CAMI I and II datasets (see Supplementary Fig. 23).

For the reclustering step, we also tried several alternative clustering methods besides weighted *k*-means, namely spectral clustering[7], agglomerative clustering and DBSCAN[8]. Weighted *k*-means outperformed all other methods (see Supplementary Fig. 24).

1.5 Evaluation of the performance of SemiBin in simulated datasets

We trained the deep learning model on the CAMI low-complexity dataset and compared it to semi-supervised SolidBin-coalign and SolidBin-CL. The original features and embedded ones from SolidBin-coalign, SolidBin-CL and SemiBin were visualized for every genome using t-SNE[9]. The siamese neural network used in SemiBin led to a better separation between genomes and better aggregation within genomes (see Fig. 2c and Supplementary Fig. 25).

When comparing the different versions of SolidBin on CAMI I datasets, in most situations, SolidBin with additional information performed worse than SolidBin-naive which showed that the semi-supervised Ncut algorithm used in SolidBin could not leverage additional information very well, perhaps due to the noise in these annotations (see Supplementary Table 2).

1.6 Applying SemiBin to real data

In the human gut dataset, SemiBin with multi-sample binning reconstructed more high-quality bins than SemiBin with single-sample binning, but these came from fewer distinct species, genera and families. This showed that multi-sample binning (which uses abundance across several samples) might lead to the recovery of more genomes from species occurring in multiple samples while overlooking rare species (see Supplementary Fig. 11).

By default, SemiBin learns an embedding model for each sample. To evaluate the generalization of this learned model, we

transferred models between different samples and environments. For this, we selected three models from each environment studied (termed as high, median and low according to the number of high-quality bins they reconstructed, see Methods) and tested their performance on 10 randomly selected samples from each environment, using single-sample binning (no overlap in training data and testing data, see Methods). Not unexpectedly, training a new model for every sample resulted in the highest number of high-quality bins while SemiBin with a pretrained model from the same environment achieved the second best result (see Supplementary Fig. 12). Nonetheless, it is noteworthy that SemiBin with a pretrained model from the same environment could still perform better than Metabat2, reconstructing at most 26.0%, 59.2% and 60.0% more high-quality bins on human gut, dog gut, and ocean testing datasets, respectively. In most situations, the pretrained model that generated the highest number of high-quality bins performed better than the model that generated median and lowest number from the same environment. Furthermore, transferring models between different environments could still improve results compared to the NoSemi version of SemiBin and in some situations, transferring across environments performed better than Metabat2 (see Supplementary Fig. 12).

The results of model transfer indicated that the siamese neural network learned the high-level or shared structure of microorganisms between environments. However, transferred models still underperformed models learned for each sample and there was a dependency on the sample used to train. Thus, we attempted to mitigate this by learning models on multiple samples simultaneously. This approach achieved the best results, while not requiring computationally-costly per-sample training (see Fig. 3 and main text).

1.7 Impact of reclustering

In the simulated datasets, the reclustering step generated significantly more high-quality bins (22.7%, 72.5% and 258.8% in CAMI I datasets and 159.2% and 50.5% in CAMI II datasets, see Supplementary Fig. 26).

In the human gut, dog gut, ocean and soil real datasets, however, the gains were smaller: with single-sample binning, there were 30/1700 (1.76%), 1/2797 (0.04%), 9/444 (2.03%), 4/103 (3.88%), while with multi-sample binning there were 105/1549 (6.78%), 216/3448 (6.26%), 44/570 (7.72%), 3/209 (1.44%) more high-quality bins resulting from the reclustering step.

1.8 Comparison of contig taxonomic annotation methods

We compared the results of SemiBin with two different contig taxonomic annotation tools: CAT[10] and MMseqs2[11,12] on the CAMI I simulated datasets as well as the human and dog gut microbiome datasets. Besides internal algorithmic differences, CAT annotates contigs to the NCBI taxonomy, while with MMseqs2 we used the GTDB as the target. We observed that, in the CAMI I datasets, CAT+NCBI performed better than MMseqs2+GTDB both in returning more accurate cannot-link constraints (see Supplementary Table 4) and more high-quality bins (see Supplementary Fig. 18). In real datasets, however, MMseqs2+GTDB performed significantly better in terms of how many high-quality bins were generated. This illustrates the perils of over-reliance on simulated benchmarks, which do not capture all the complexity of real data. Therefore, we chose

MMseqs2 and the GTDB as the default contig annotation tool and taxonomy in SemiBin (although it can easily be replaced by the user).

1.9 Evaluation of SemiBin when removing related genomes from the GTDB

To demonstrate that SemiBin's performance was not overestimated in the CAMI datasets due to the presence of very similar genomes in the annotation database, we removed related genomes from the GTDB at different taxonomic ranks and then annotated the contigs to obtain the cannot-link constraints. First, we annotated the source genomes from CAMI I datasets using GTDB-Tk[13] (version 1.4.1, using classify_wf workflow with default parameters) and then excluded genomes that matched the corresponding species, genus, family, order, class and phylum from GTDB reference genomes using MMseqs2 (13.45111, 'filtertaxseqdb' command). We then annotated the contigs with the corresponding databases and trained the model from the corresponding cannot-link constraints. The results showed that SemiBin did not rely on the presence of closely related genomes in the reference databases, as we observed only a very minor loss in the number of high-quality bins recovered, even when excluding all genomes that matched the same phylum from the GTDB (see Supplementary Fig. 27).

1.10 Analysis of *Bacteroides vulgatus* strains

Comparing the average nucleotide identity (ANI) of 50 *B. vulgatus* bins (49 recovered from our data, and one reference genome, GCF000012825), we found that the ANI of *B. vulgatus* strains within the dog gut microbiome (average 99.4%) was significantly higher than those within human gut microbiome (average 98.8%) (see Supplementary Fig. 17a). To further explore the diversity of the *B. vulgatus* strains, we predicted and annotated protein-coding sequences (CDS) with Prokka[14] and built the pangenome with Roary[15] using a threshold of 95% (nucleotide identity) for gene similarity. We identified a total of 15,382 genes, of which only 458 are core genes shared between all 50 MAGs. These core genes were used to infer a maximum likelihood phylogenetic tree using IQTREE[16] (see Fig. 6). It was clear that MAGs from the dog gut tend to cluster together. The same results can be found when performing principal component analysis (PCA) based on the presence/absence of genes of the 50 *B. vulgatus* strains (see Supplementary Fig. 17b).

We observed that a total of 13 genes were differentially present in dog and human gut strains (see Fig. 6 and main text). Using the Global Microbial Gene Catalog (GMGCv1[17]), which contains 762 *B. vulgatus* high-quality strains from human gut microbiomes (recovered using Metabat2), for external validation, we verified that 7 of them were still significant (see Supplementary Fig. 28). Furthermore, in the maximum likelihood phylogenetic tree generated with GMGCv1 strains, MAGs from dog guts still clustered together (see Supplementary Fig. 28).

2 Supplementary Figures and Tables

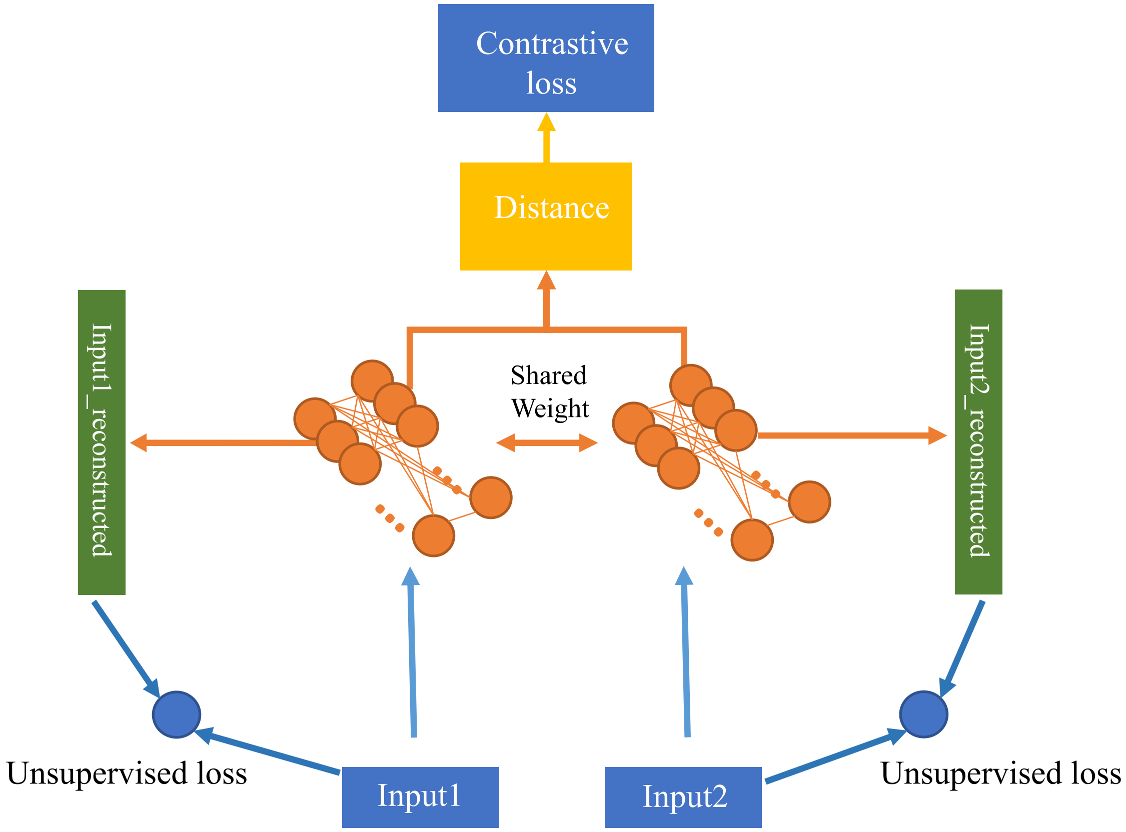

Supplementary Fig 1. Semi-supervised siamese neural network model used in SemiBin. A shared-weight neural network takes as input a pair (input1, input2). Inputs can be *k*-mer frequencies and the abundance distribution ($n \geq 5$) or just the *k*-mer frequencies ($n < 5$). During training, the unsupervised loss and the contrastive loss are optimized at the same time. After training, the embedding of the inputs can be used for clustering.

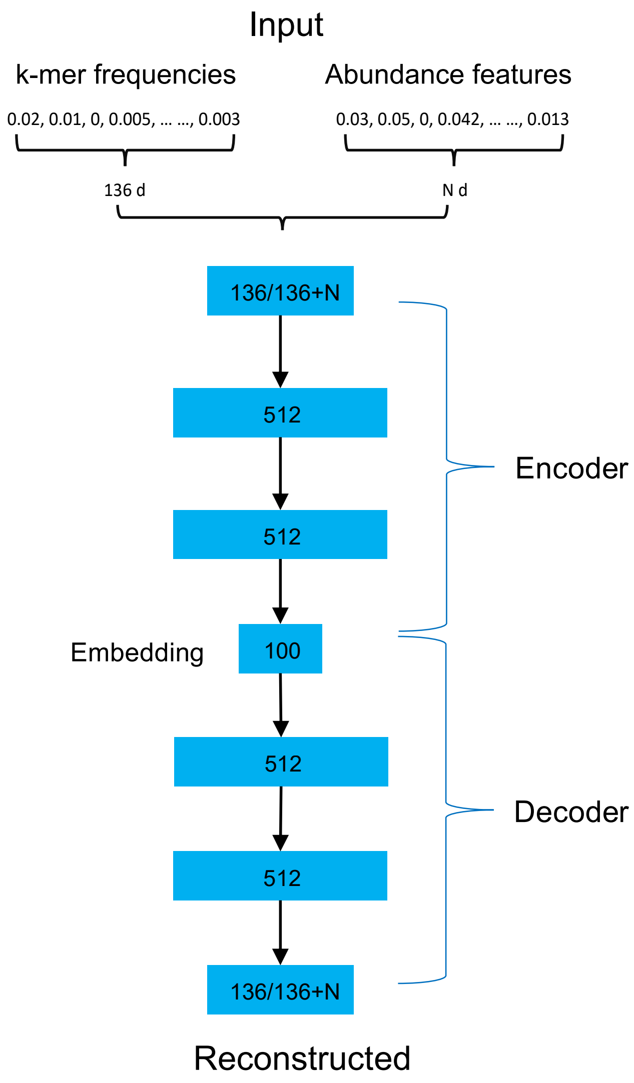

Supplementary Fig 2. Structure of the semi-supervised siamese neural network model used in SemiBin. The inputs to the model are the *k*-mer frequencies and the abundance features (depending on the number of samples used in the binning, see Methods). The number in the box is the number of neurons in every layer. The neural network used in SemiBin is a shared-weight autoencoder. An encoder network encodes the inputs to 100 dimension features and a decoder network reconstructs the original inputs. After training, the 100 dimensional embeddings are used in the binning.

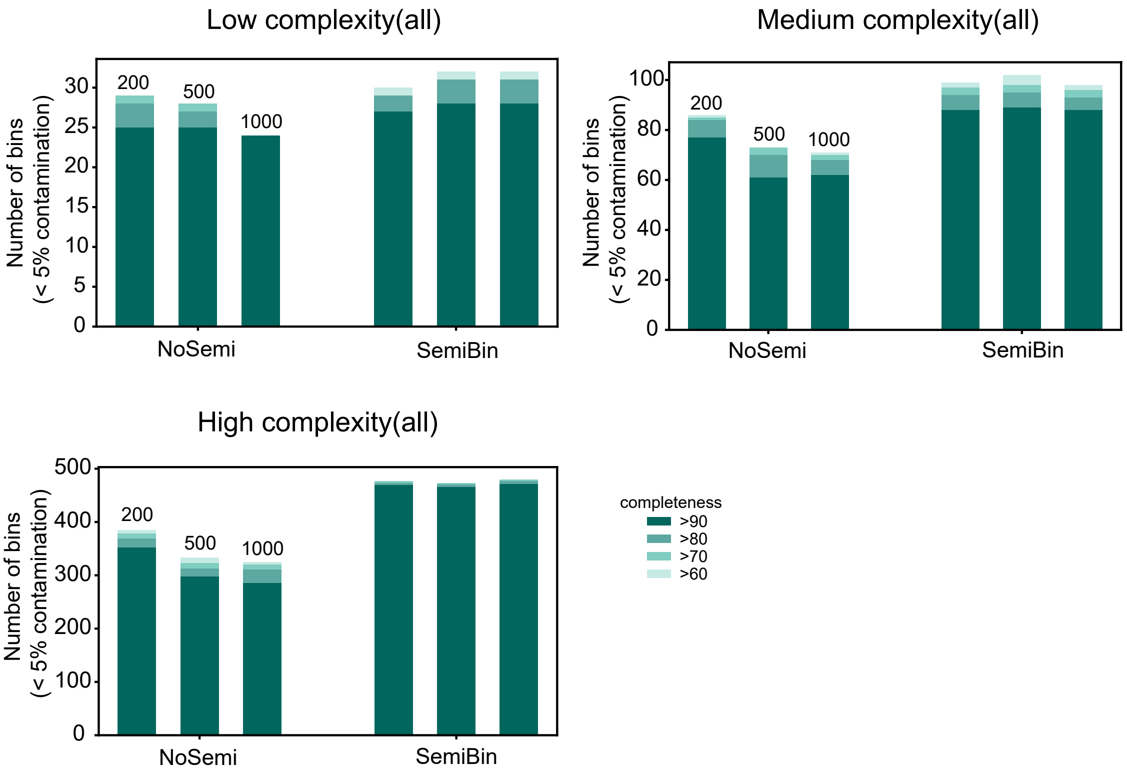

Supplementary Fig 3. Semi-supervised learning in SemiBin significantly improved binning results. We compared SemiBin to the NoSemi version (removing the semi-supervised learning component in SemiBin) to show the performance of the semi-supervised learning in SemiBin. Shown are the numbers of reconstructed genomes (with varying completeness and smaller than 5% contamination) for low complexity dataset, medium complexity dataset and high complexity dataset.

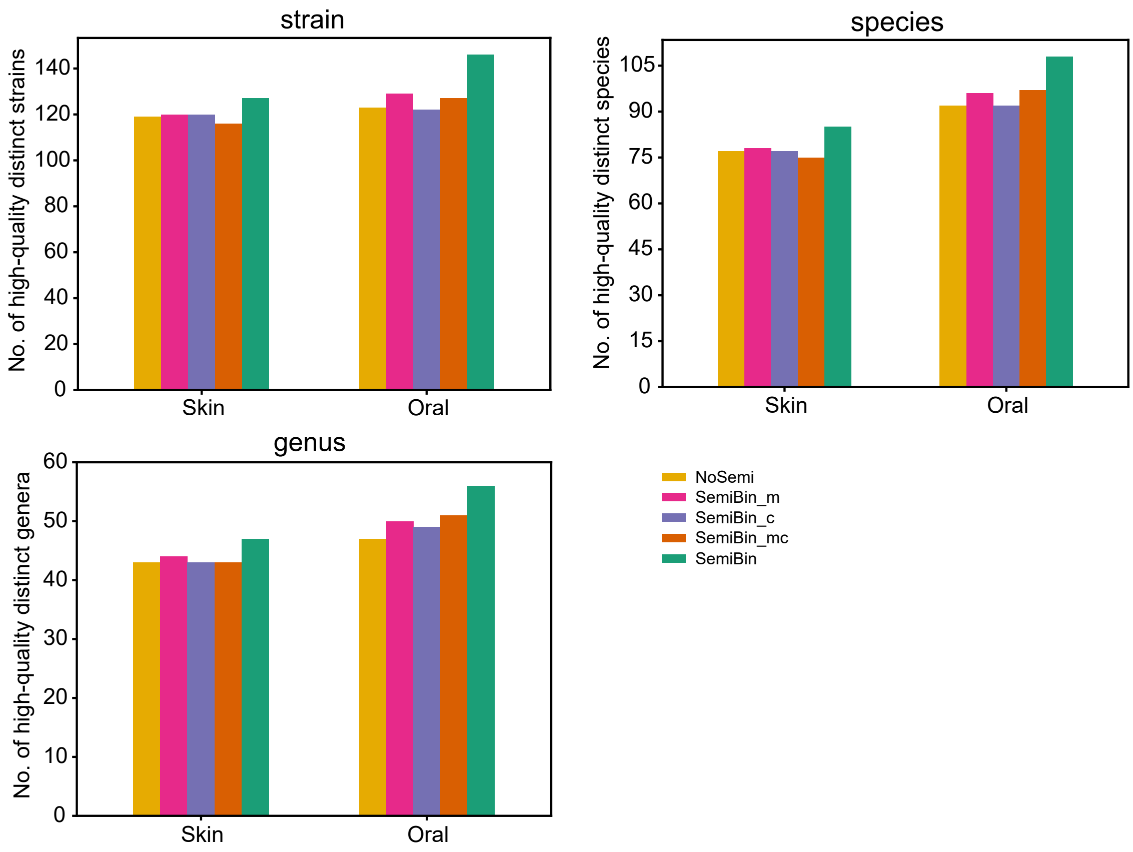

Supplementary Fig 4. The deep siamese neural network in SemiBin learnt the underlying structure of the environment in CAMI II datasets. To evaluate that the semi-supervised learning model could learn the underlying structure of the environment (not just reproduce the must-link and cannot-link constraints), we compared SemiBin to the NoSemi version (removing the semi-supervised learning component in SemiBin), SemiBin_m (directly using must-link constraints to generate the sparse network for clustering, no semi-supervised learning), SemiBin_c (directly using cannot-link constraints to generate the sparse network for clustering, no semi-supervised learning), and SemiBin_mc (directly using must-link and cannot-link constraints to generate the sparse network for clustering, no semi-supervised learning) (see Methods). Shown are the numbers of high-quality distinct strains, species and genera in the Skin and Oral datasets.

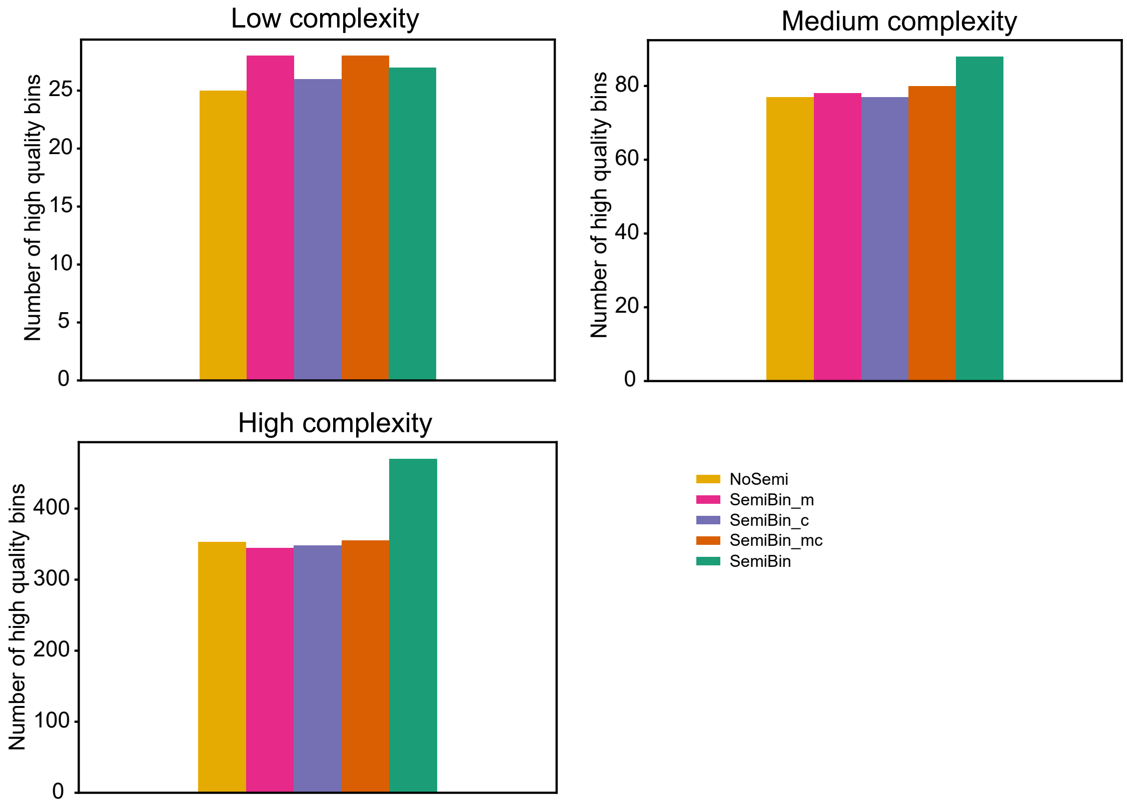

Supplementary Fig 5. The deep siamese neural network in SemiBin learnt the underlying structure of the environment in CAMI I datasets. Shown are the numbers of high-quality bins in low complexity, medium complexity and high quality datasets from CAMI I. The methods used here are the same as those in Supplementary Fig. 4.

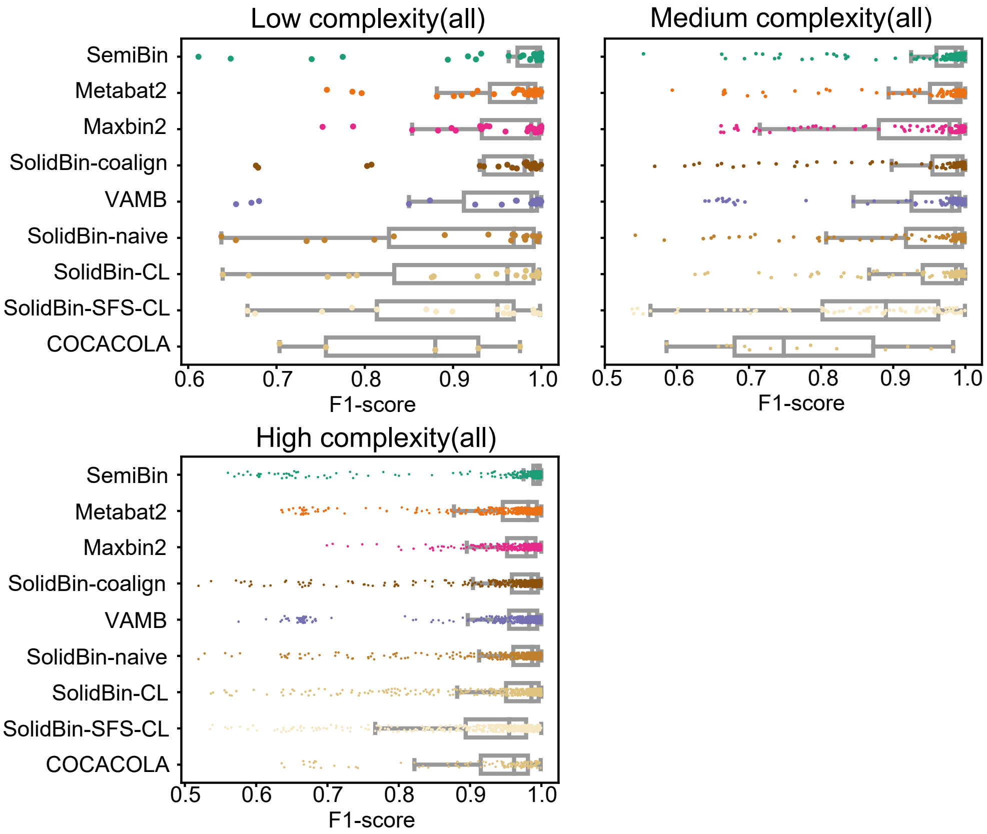

Supplementary Fig 6. SemiBin outperformed other binners on CAMI I datasets with single-sample and co-assembly binning. Shown are the F1-score distributions of bins (completeness $\geq$ 50%; contamination $\geq$ 50%) reconstructed from low, medium and high complexity datasets of CAMI I with single-sample and co-assembly binning. The number of bins from these methods for the low, medium and high complexity datasets are SemiBin (n = 36, 117 and 537), Metabat2 (n = 32, 105 and 468), Maxbin2 (n = 30, 97 and 296), SolidBin-coalign (n = 21, 98 and 505), VAMB (n = 20, 78 and 445), SolidBin-naive (n = 20, 103 and 522), SolidBin-CL (n = 19, 92 and 518), SolidBin-SFS-CL (n = 17, 87 and 506) and COCACOLA (n = 5, 14 and 129). For the box plots, the center line is the median of all values, the lower and upper bounds of the box correspond to 25th and 75th percentiles and the lower and upper of the whiskers are the minimum and maximum values.

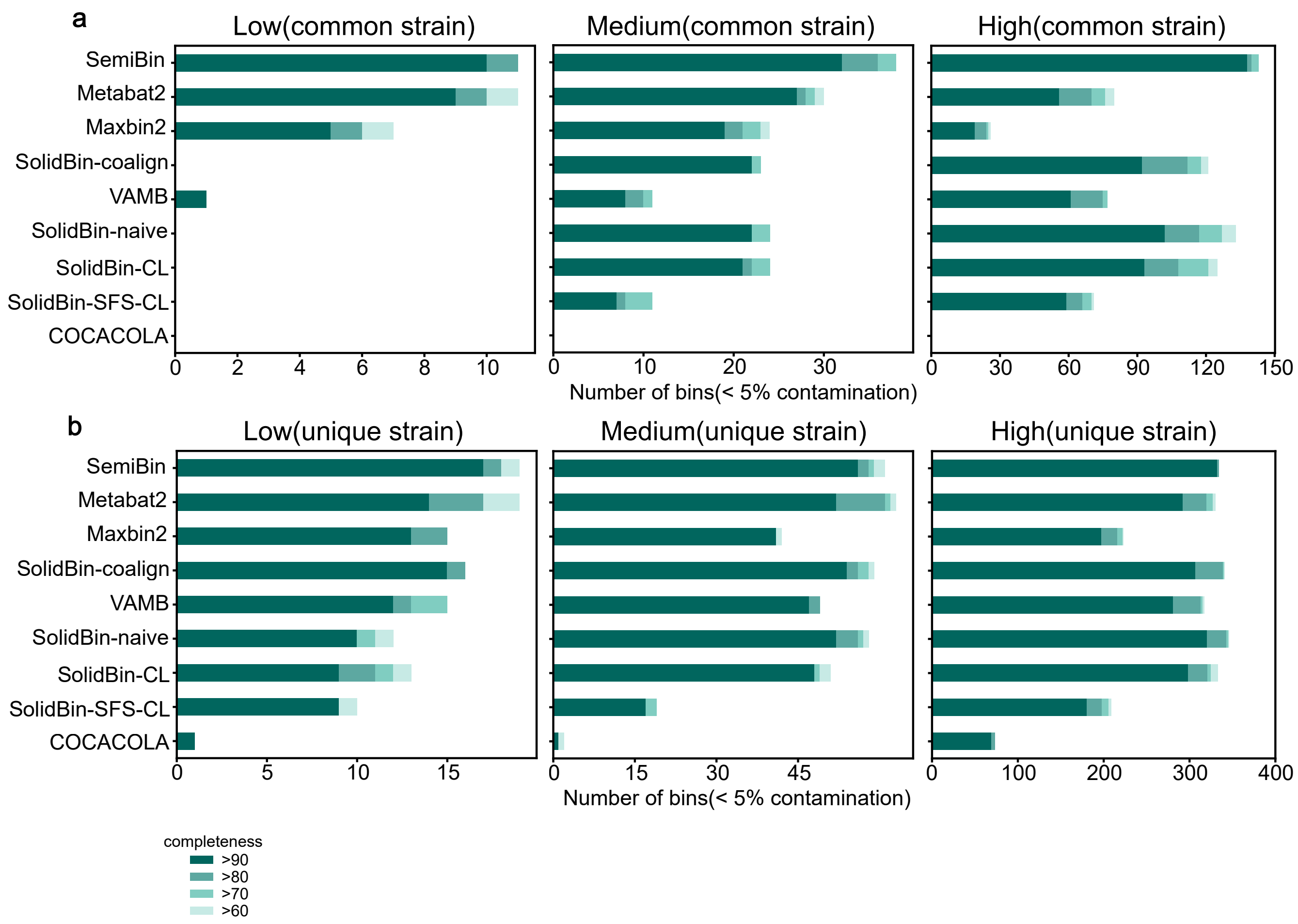

Supplementary Fig 7. SemiBin outperformed other binners in CAMI I datasets. Genomes in CAMI I datasets are defined as either common strains or unique strains. Common strains are defined as genomes with an ANI (average nucleotide identity) $\geq 95\%$ to the most similar genomes in the environment and unique strains are defined as genomes with $< 95\%$ ANI value to every other genome. Shown are the numbers of reconstructed genomes per method above varying completeness and contamination $< 5\%$ for **a,** three datasets considering common strains; and **b,** three datasets considering unique strains. SemiBin reconstructed more high-quality bins (considering all strains, common strains and unique strains), especially for common strains which is a big challenge for binning in an environment with multiple strains.

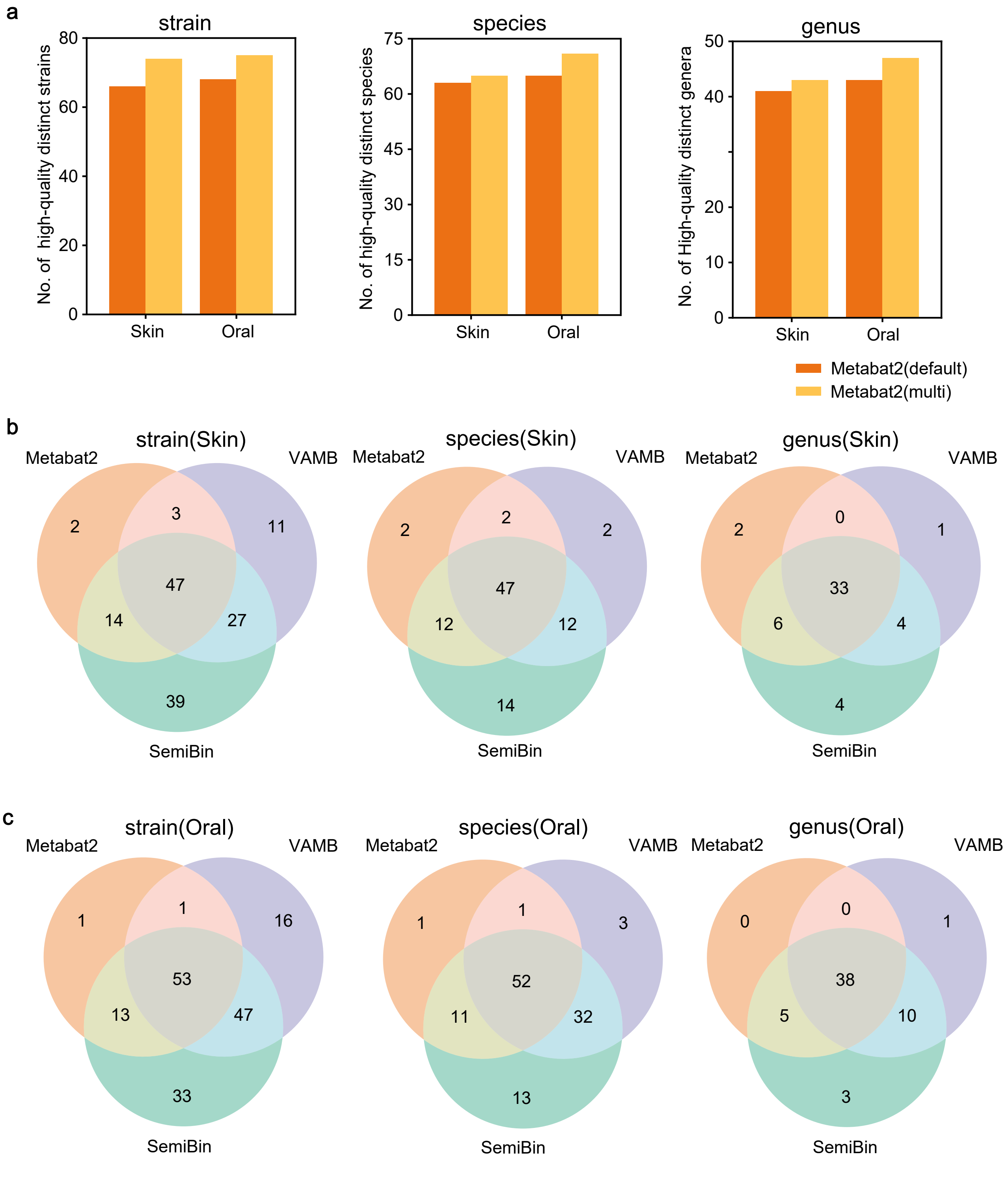

Supplementary Fig 8. SemiBin outperformed Metabat2 and VAMB in CAMI II datasets with multi-sample binning. **a,** The comparison of Metabat2 with single-sample binning (Metabat2(default)) and our adapted multi-sample binning (Metabat2(multi)). The adaptation led to only modest improvements. **b,** and **c,** Shown are the overlaps of the reconstructed distinct high-quality strains, species and genus for the Skin and Oral datasets. SemiBin reconstructed more distinct high-quality strains, species and genera compared to VAMB and Metabat2.

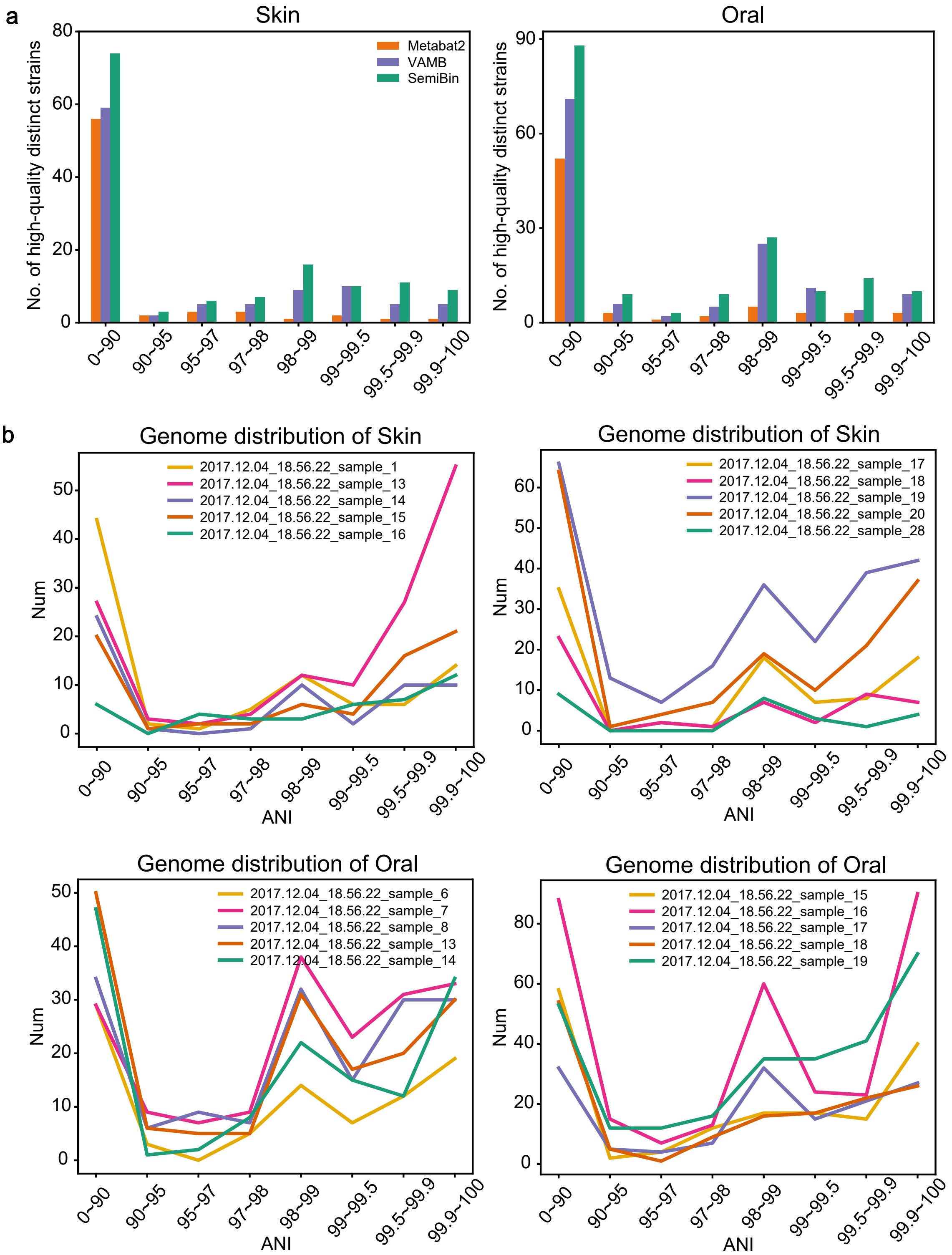

Supplementary Fig 9. SemiBin outperformed Metabat2 and VAMB across almost all ANI intervals in CAMI II datasets. We stratified the datasets according to the ANI value of each genome to the most similar genome present in the same sample. We calculated the number of distinct high-quality strains in every interval. Shown are **a,** the numbers of reconstructed distinct high-quality strains in every interval, and **b,** the genome distributions according to the ANI values for every sample of Skin and Oral datasets.

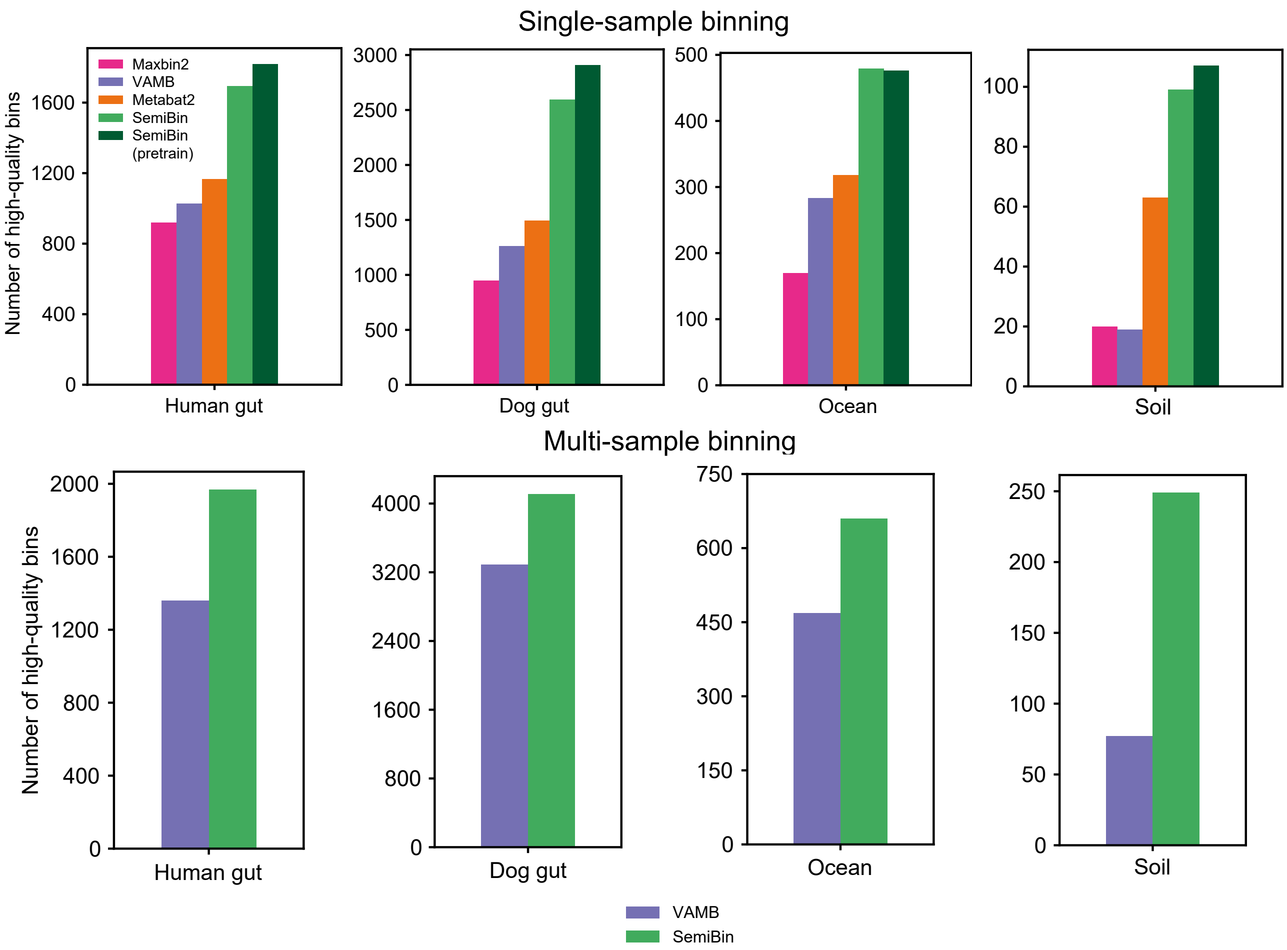

Supplementary Fig 10. SemiBin outperformed other binners in real datasets with single-sample and multi-sample binning evaluated by CheckM. A high-quality bin is defined as a bin with completeness > 90% and contamination < 5% evaluated by CheckM[18] (cf. Fig. 3b, which shows results including chimera filtering with GUNC[19]). Shown are the numbers of high-quality bins generated by Maxbin2, VAMB, Metabat2, SemiBin and SemiBin(pretrain) with single-sample binning and VAMB and SemiBin with multi-sample binning in the human gut, dog gut, ocean and soil datasets. Based on results from CheckM, SemiBin(pretrain) reconstructed 55.7%, 94.6%, 49.7% and 69.8% more high-quality bins than Metabat2 with single-sample binning and 44.7%, 25.0%, 41.0% and 223.4% more high-quality bins than VAMB with multi-sample binning in the human gut, dog gut, ocean and soil datasets, respectively.

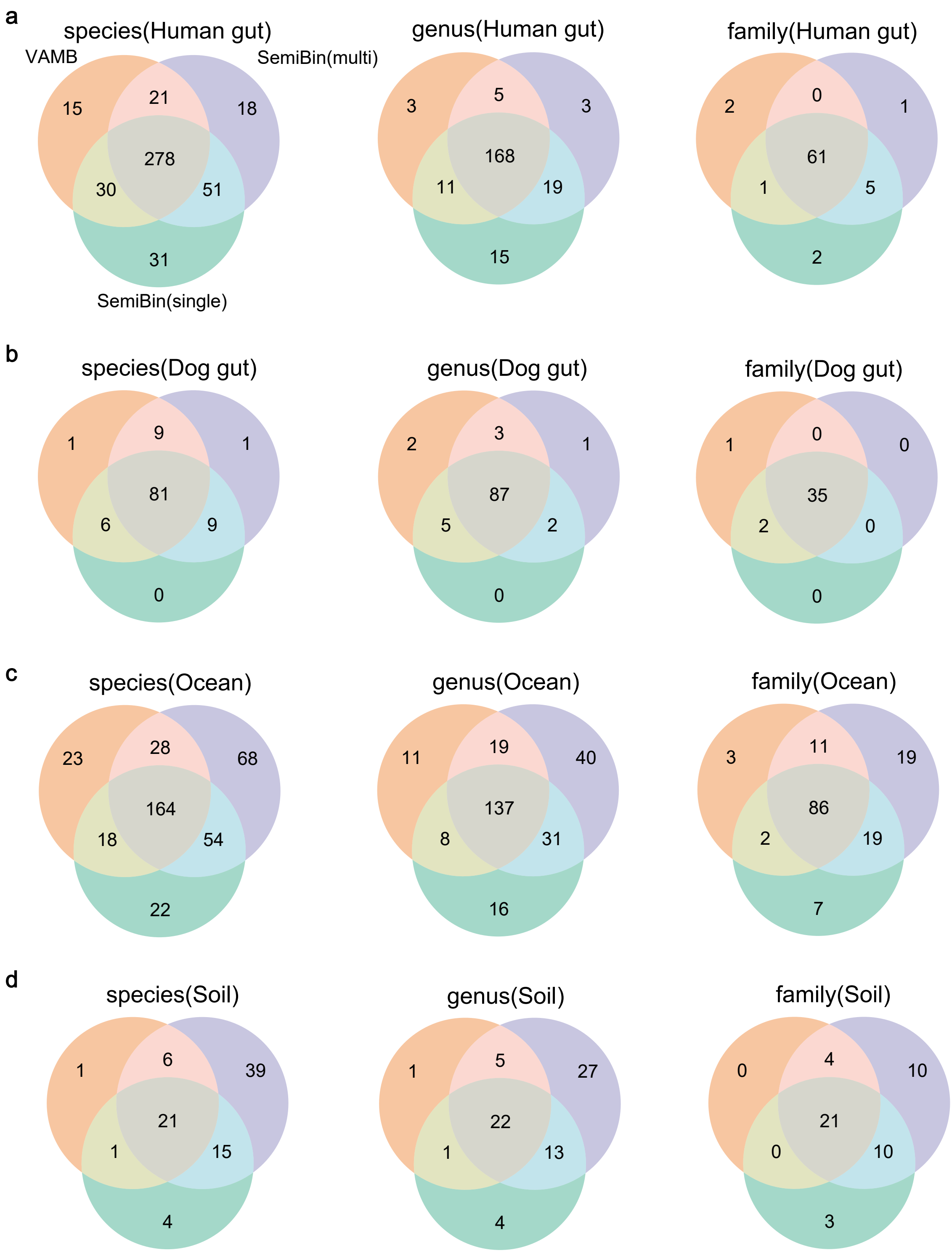

Supplementary Fig 11. SemiBin reconstructed more high-quality distinct species, genera and families with multi-sample binning. We compared the number of high-quality distinct species, genera and families returned by VAMB with multi-sample binning, SemiBin(multi) (SemiBin with multi-sample binning) and SemiBin(single) (SemiBin with single-sample binning) for **a,** human gut; **b,** dog gut; **c,** ocean and **d,** soil dataset.

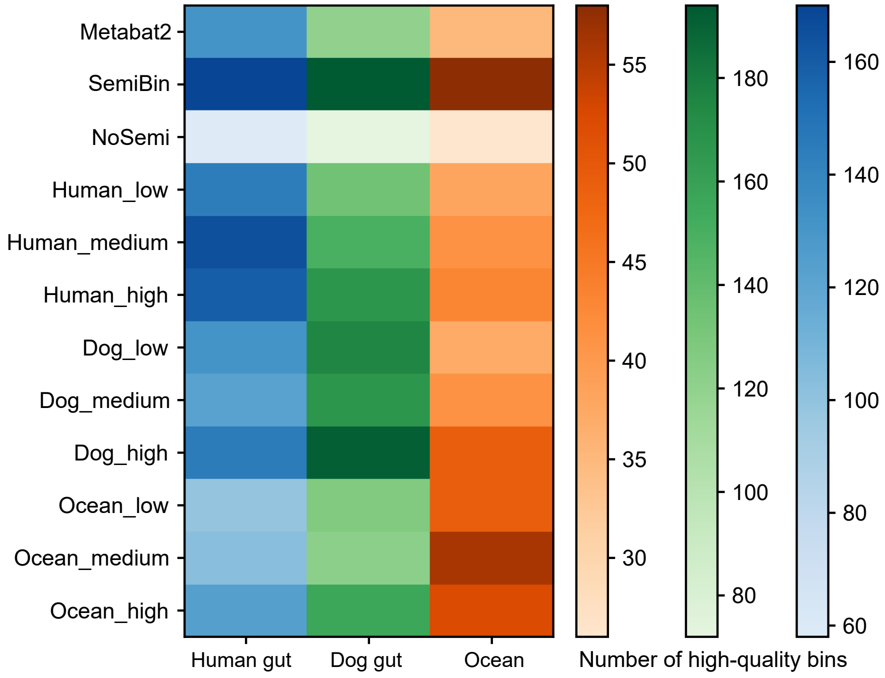

Supplementary Fig 12. SemiBin with pretrained model trained from one sample got competitive binning results. We transferred the learned semi-supervised models from one sample between the human gut, dog gut and ocean datasets. We chose three models from samples that reconstructed the highest, median and lowest number of high-quality bins for each environment, and termed these models as human_high, human_medium, human_low, dog_high, dog_medium, dog_low, ocean_high, ocean_medium, ocean_low. For every environment, we randomly chose 10 samples that were not used before as testing sets (no overlap in training samples and testing samples). The transfer results compared to Metabat2, original SemiBin and NoSemi version are shown as the number of high-quality bins for each environment (the darker the color, the higher the number of high-quality bins).

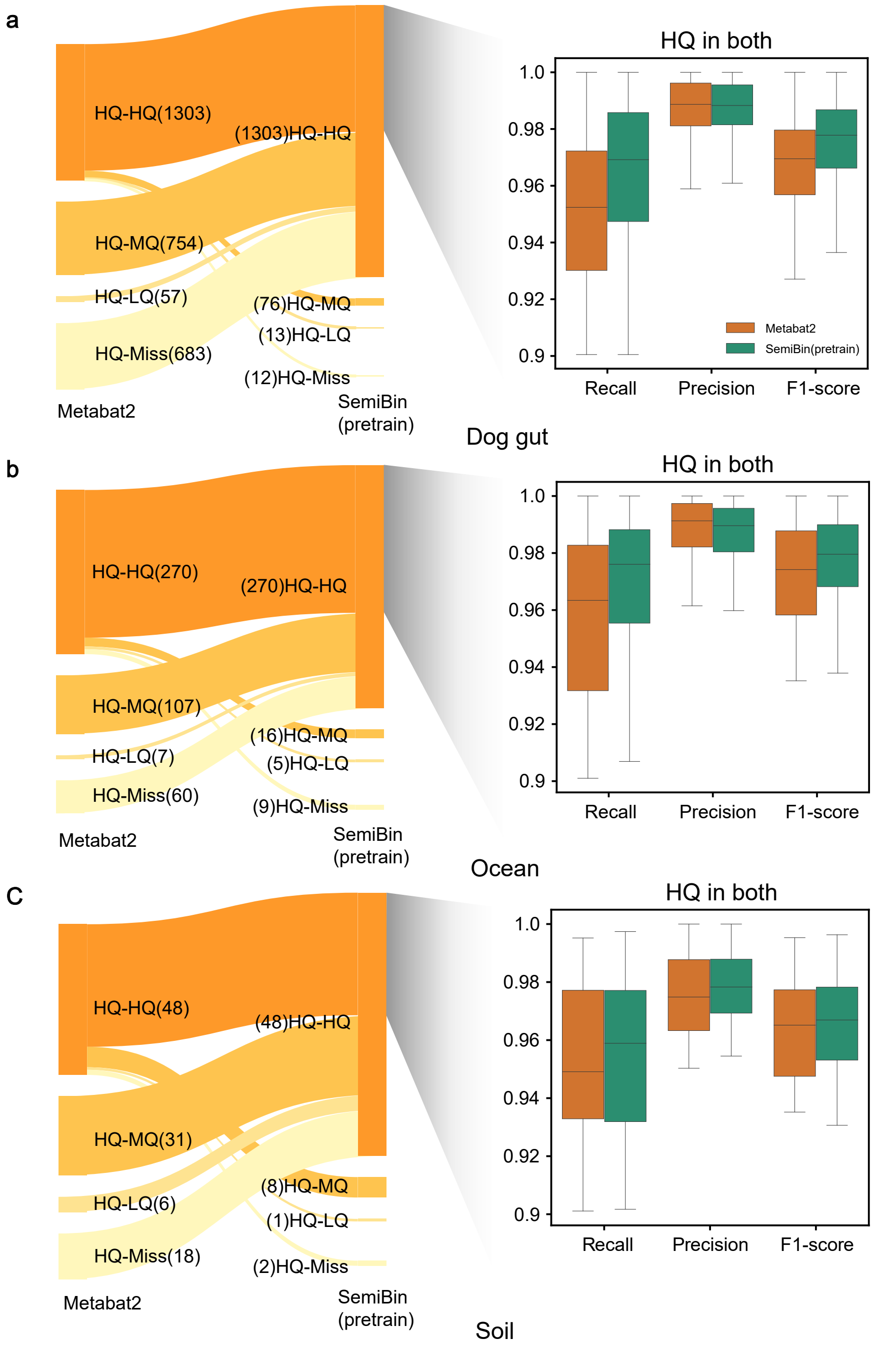

Supplementary Fig 13. SemiBin(pretrain) reconstructed more and better high-quality bins compared to Metabat2 in dog gut, (a) ocean (b) and soil datasets (c). Results here are qualitatively similar to results in the human gut datasets (see Fig. 4). In the dog gut, ocean datasets, within bins that are high-quality in both binners, SemiBin(pretrain) significantly achieved higher completeness ($P = 7.338 \cdot 10^{-110}$ (n = 129); $P = 5.266 \cdot 10^{-20}$ (n = 109)) and F1-score ($P = 1.107 \cdot 10^{-110}$ (n = 129); $P = 4.331 \cdot 10^{-15}$ (n = 109)), with slightly increase in contamination ($P = 0.165 > 0.05$ (n = 129); $P = 3.953 \cdot 10^{-05}$ (n = 109)). In the soil dataset, SemiBin(pretrain) achieved similar completeness ($P = 0.106$ (n = 101)), slightly less contamination ($P = 0.052$ (n = 101)) and slightly better F1-scores ($P = 0.008$ (n = 101)). All *P*-values were computed using Wilcoxon signed rank test, two-sided null hypothesis. For the box plots, the center line is the median of all values, the lower and upper bounds of the box correspond to 25th and 75th percentiles and the lower and upper of the whiskers are the minimum and maximum values.

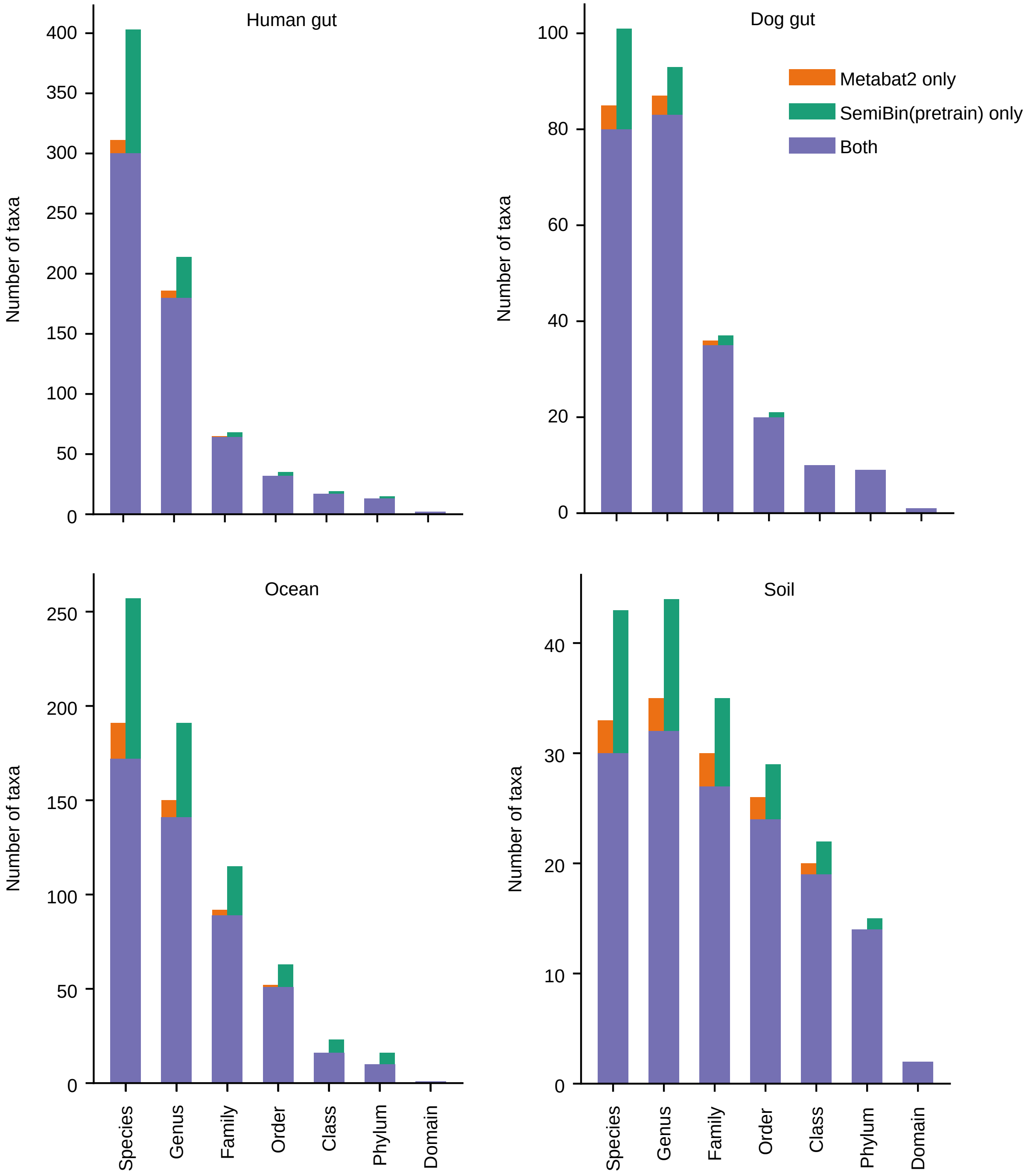

Supplementary Fig 14. SemiBin(pretrain) returns a larger taxonomic diversity at all levels compared to Metabat2 with single-sample binning in real datasets. We annotated the high-quality bins from SemiBin(pretrain) and Metabat2 with GTDB-Tk. Shown are the numbers of distinct taxa at the species, genus, family, order, class, phylum and domain level.

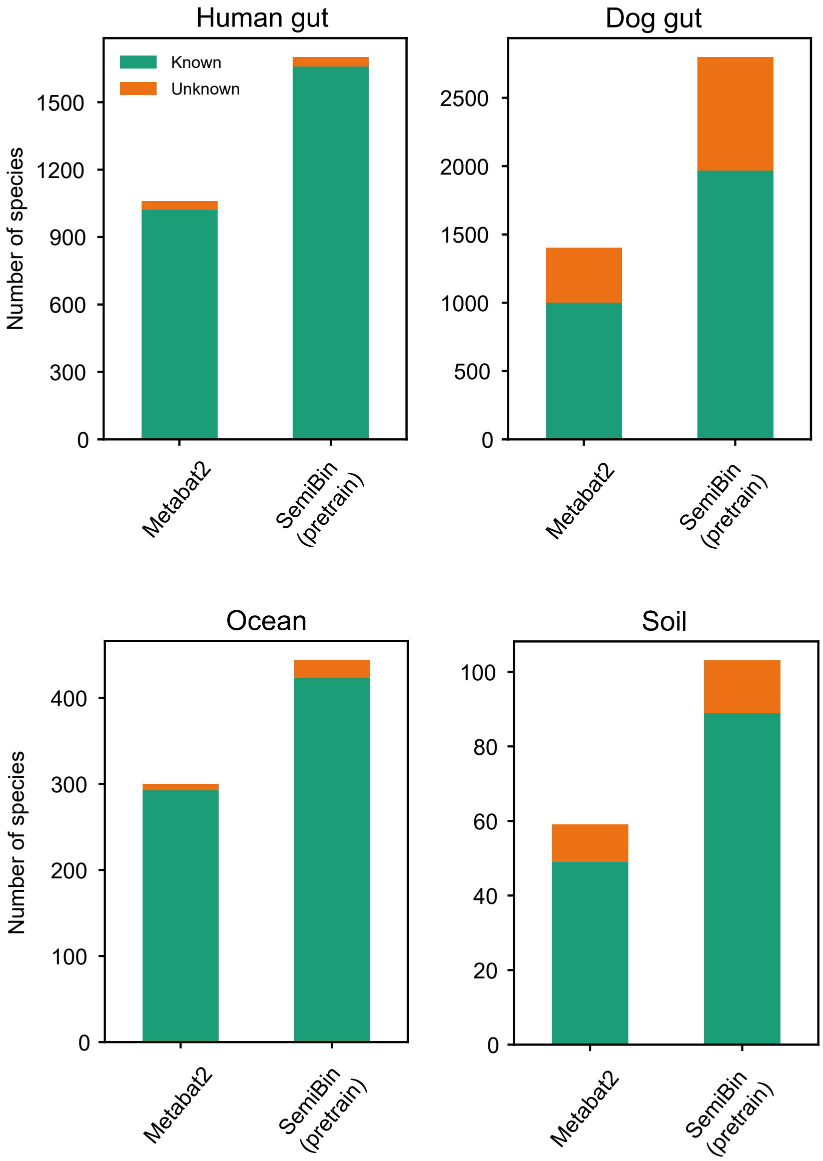

Supplementary Fig 15. SemiBin(pretrain) reconstructed more known and unknown species. We annotated the high-quality bins from SemiBin(pretrain) and Metabat2 with GTDB-Tk. Shown are the numbers of known and unknown species.

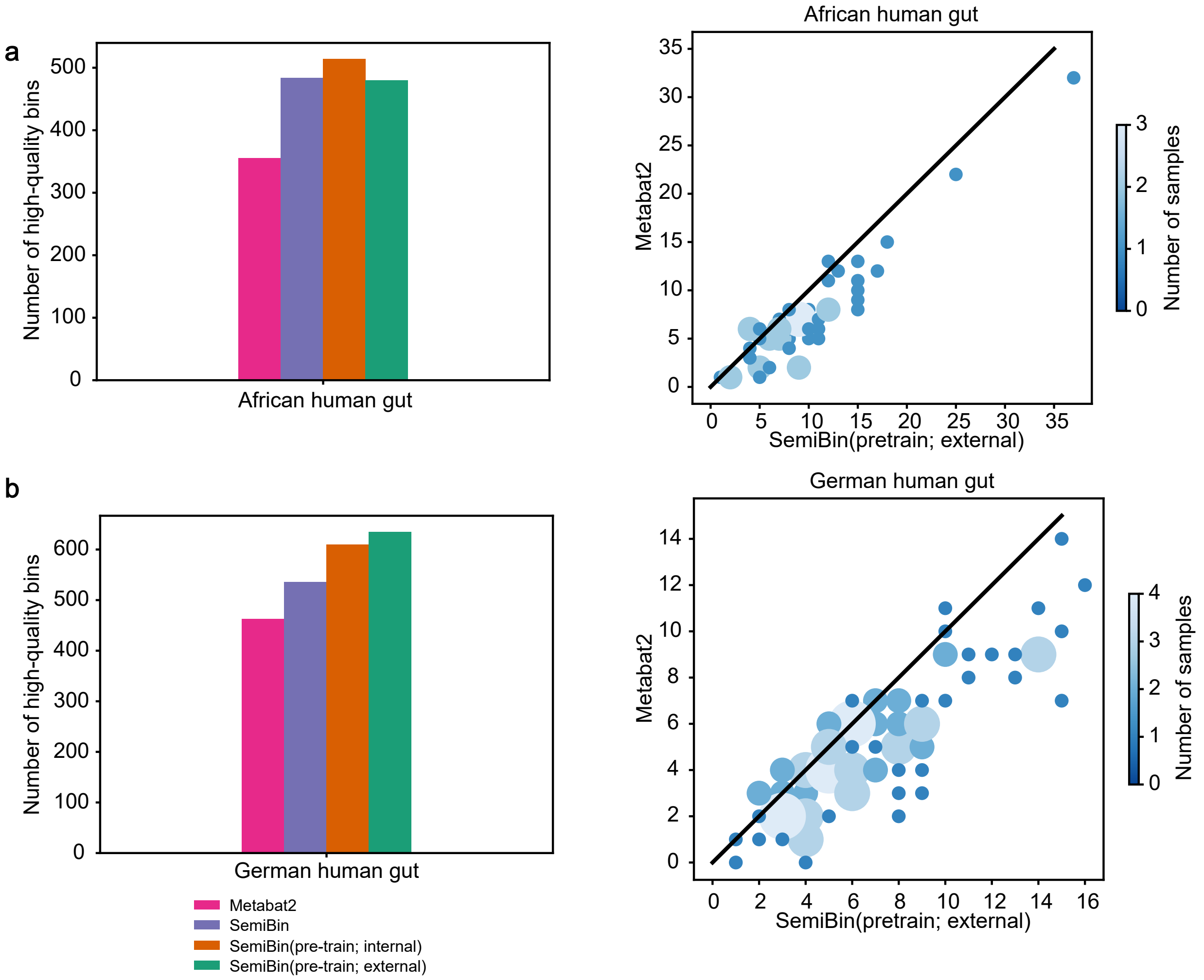

Supplementary Fig 16. SemiBin with the pretrained model outperformed Metabat2 on two hold-out human gut datasets from African and German populations. We transferred the pretrained model from the human gut dataset used previously to two hold-out human gut datasets (one from an African non-Westernized population and another from a German population). We also benchmarked Metabat2, SemiBin and SemiBin with a model trained on the hold-out datasets (using 20 samples). (SemiBin(pretrain; internal): SemiBin with a pretrained model from the same dataset as the one where the evaluation is carried out; SemiBin(pretrain; external): SemiBin with the pretrained model from the human gut dataset used in Fig. 3b. Shown are the number of high-quality bins (**left**) and the number of samples with the particular combination of the number of high-quality bins generated from Metabat2 (y-axis) and SemiBin(pretrain; external) (x-axis) (**right**). **a,** African human gut; **b,** German human gut.

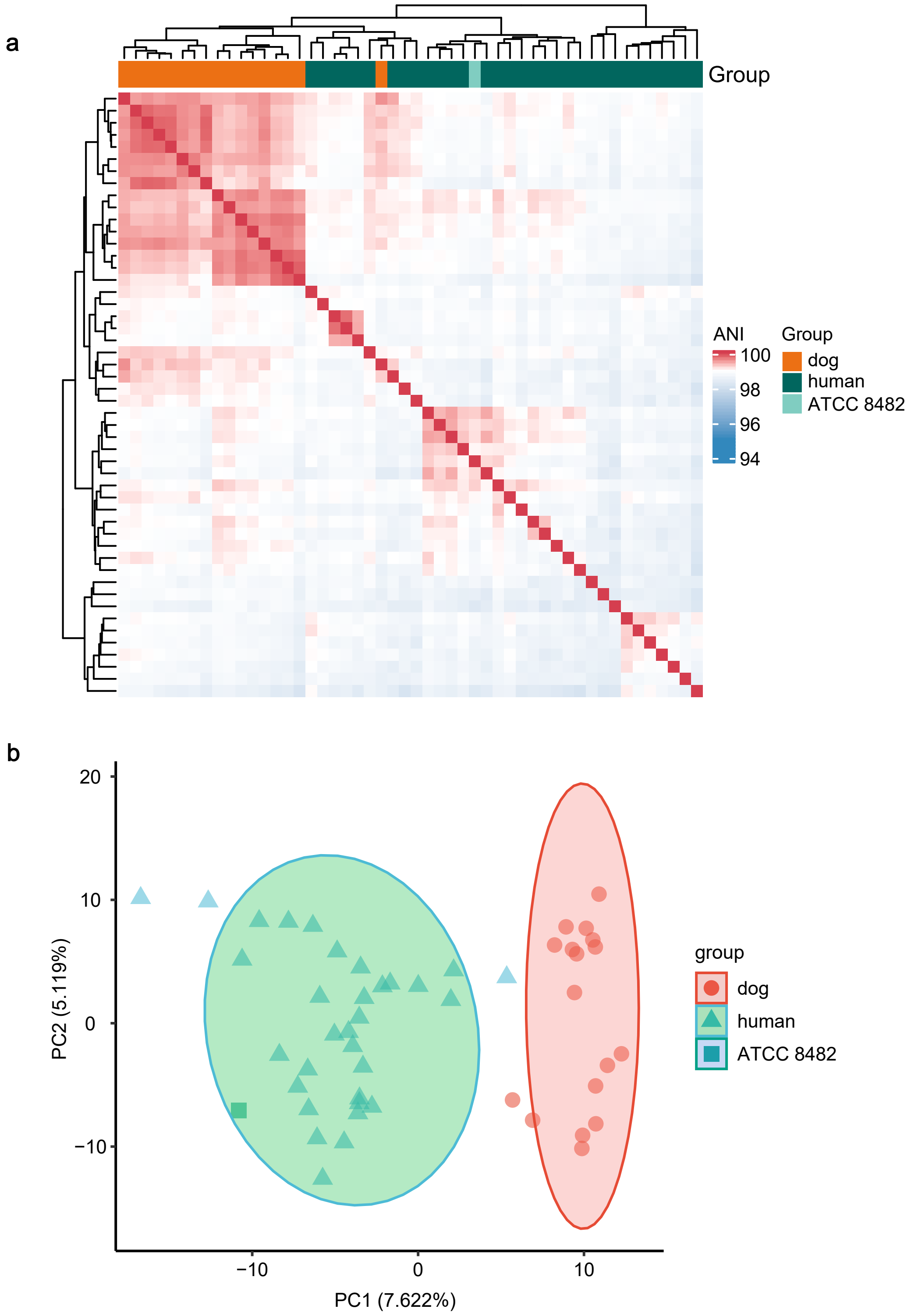

Supplementary Fig 17. ***B. vulgatus*** **bins from the dog gut clustered separately from those of the human gut. a,** Heatmap representing the average nucleotide identity (ANI) similarity of the 49 strains of *B. vulgatus* studied and a type strain *B. vulgatus* ATCC 8482. The color scheme varies from high ANI similarity (red) to low ANI similarity (blue) of the strains analyzed. **b,** Principal component analysis (PCA) based on whole presence/absence genes.

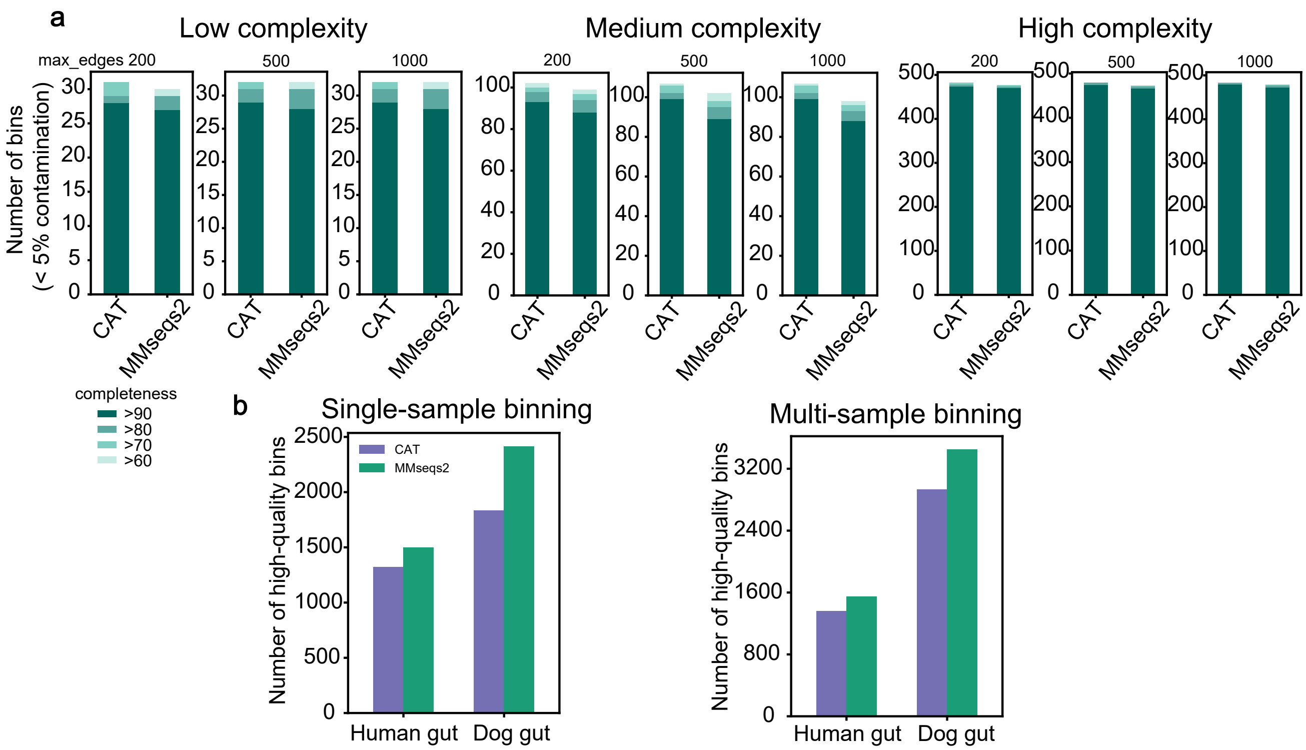

Supplementary Fig 18. SemiBin with a better reference got better binning results. We compared the results of SemiBin with contig annotations by CAT (using NCBI taxonomy) and MMseqs2 (using GTDB taxonomy). Shown are **a,** the number of reconstructed genomes (with varying completeness and smaller than 5% contamination) on CAMI I datasets and **b,** the number of high-quality bins on human gut and dog gut real datasets.

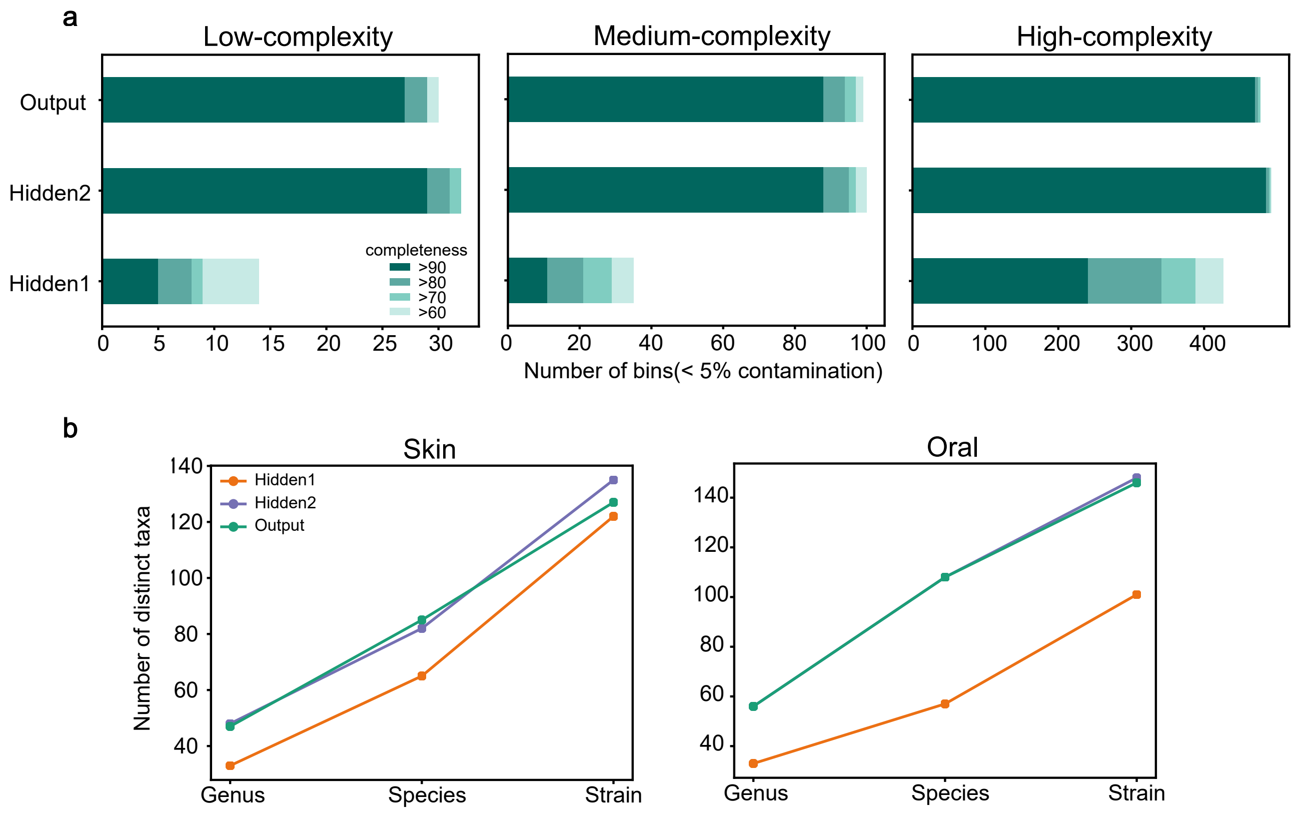

Supplementary Fig 19. The influence of different embeddings to the binning results in simulated datasets. SemiBin uses a shared-weight autoencoder (see Supplementary Fig. 2). For the encoder component, there are two hidden layers and one output layer. The standard SemiBin pipeline uses the features from the output layer (100 dimensions). Here, we benchmarked using the features from the first and second hidden layers (512 dimensions) as alternatives. Features from the second hidden layer and the output layer showed similar results, while using features from the first hidden layer resulted in significantly worse results, indicating that the first layer did not learn enough information for binning. **a,** Shown are the numbers of reconstructed genomes with varying completeness and contamination < 5% on CAMI I datasets. **b,** Shown are the numbers of high-quality distinct genera, species and strains on CAMI II datasets.

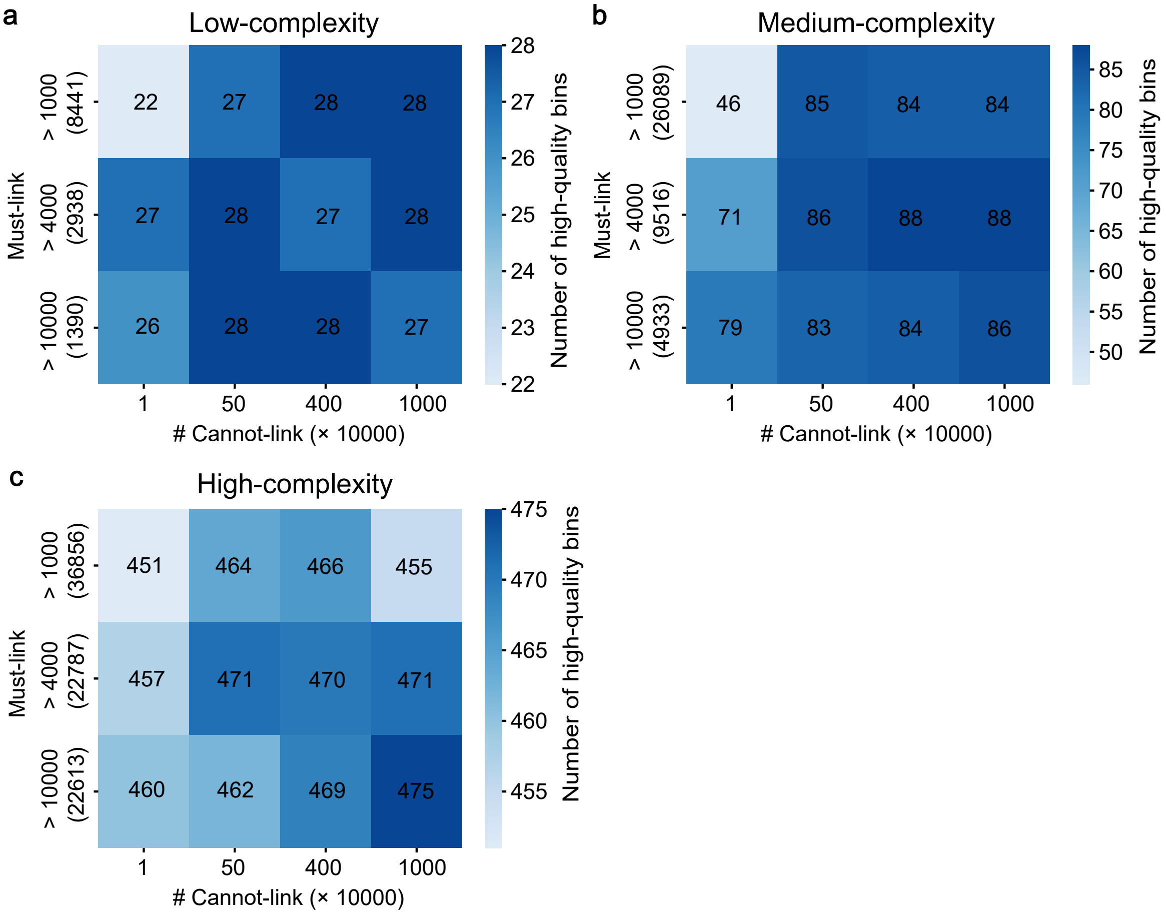

Supplementary Fig 20. SemiBin can be robust to different numbers of must-link and cannot-link constraints. Shown are the numbers of high-quality bins from different numbers of must-link and cannot-link constraints. **a,** low-complexity, **b,** medium-complexity and **c,** high-complexity dataset.

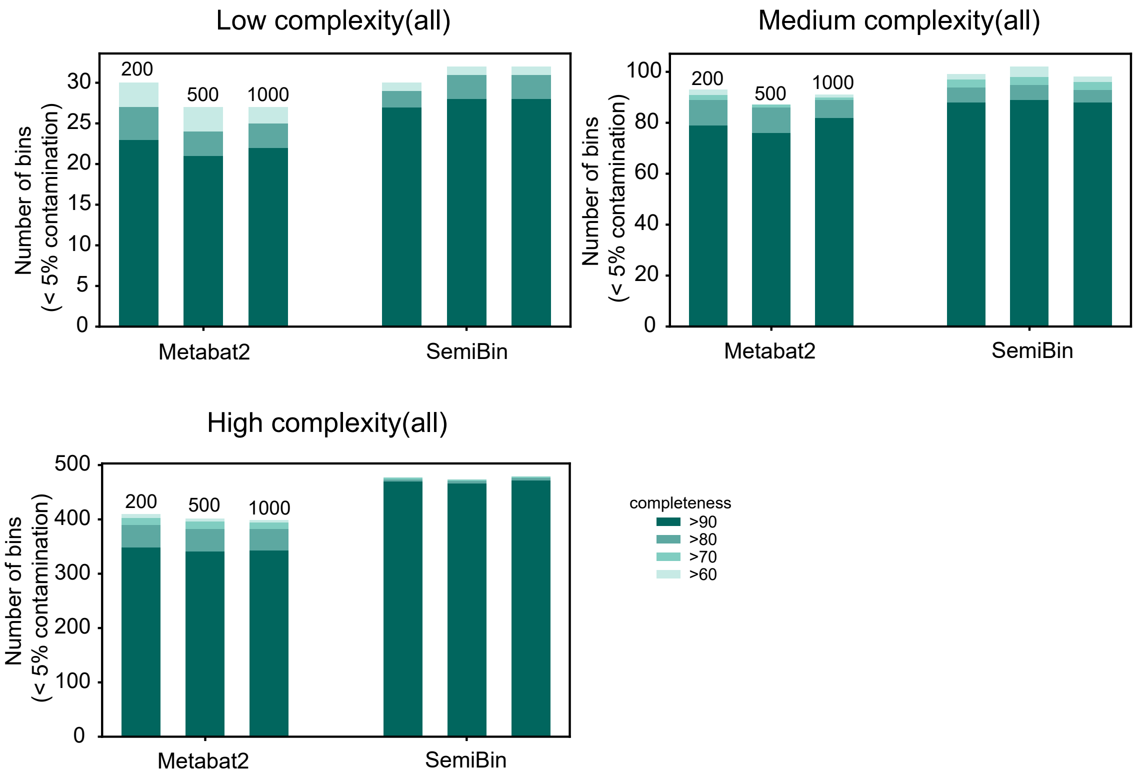

Supplementary Fig 21. SemiBin outperformed Metabat2 with different max_edges settings (200, 500 and 1000) in CAMI I datasets. We investigated the influence of different values (200, 500, 1000) of the parameter max_edges used in SemiBin and Metabat2. SemiBin outperformed Metabat2 in all situations. Shown are the numbers of reconstructed genomes with varying completeness and contamination < 5%. **a,** low complexity dataset; **b,** medium complexity dataset; and **c,** high complexity dataset.

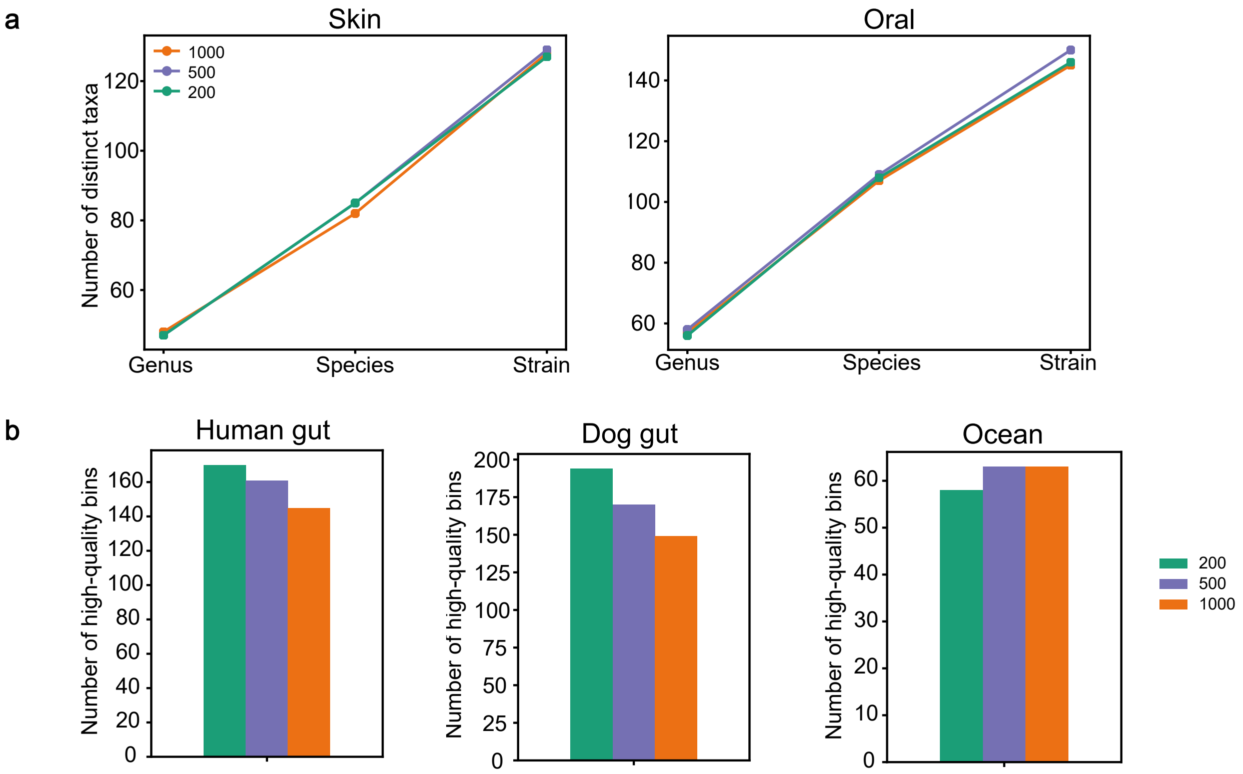

Supplementary Fig 22. The impact of changing the value of the max_edges parameter to the binning results. Shown are the binning results with different values for the max_edges parameter. **a,** The numbers of high-quality distinct genera, species and strains on CAMI II and **b,** the numbers of high-quality bins on real datasets (10 randomly-chosen samples).

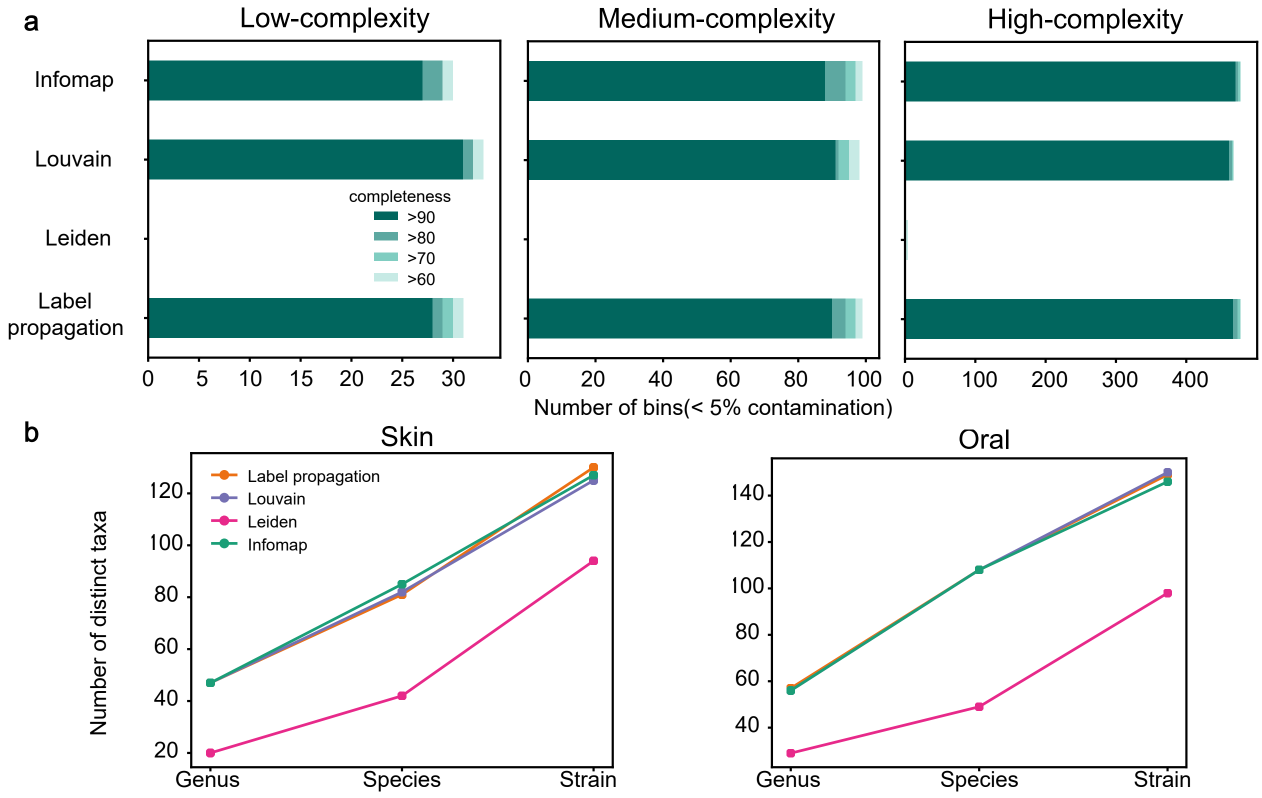

Supplementary Fig 23. Different community detection methods return similar results. Shown are the binning results using different community detection methods. **a,** The number of reconstructed genomes with varying completeness and contamination < 5% on CAMI I datasets and **b,** The numbers of high-quality distinct genera, species and strains on CAMI II datasets.

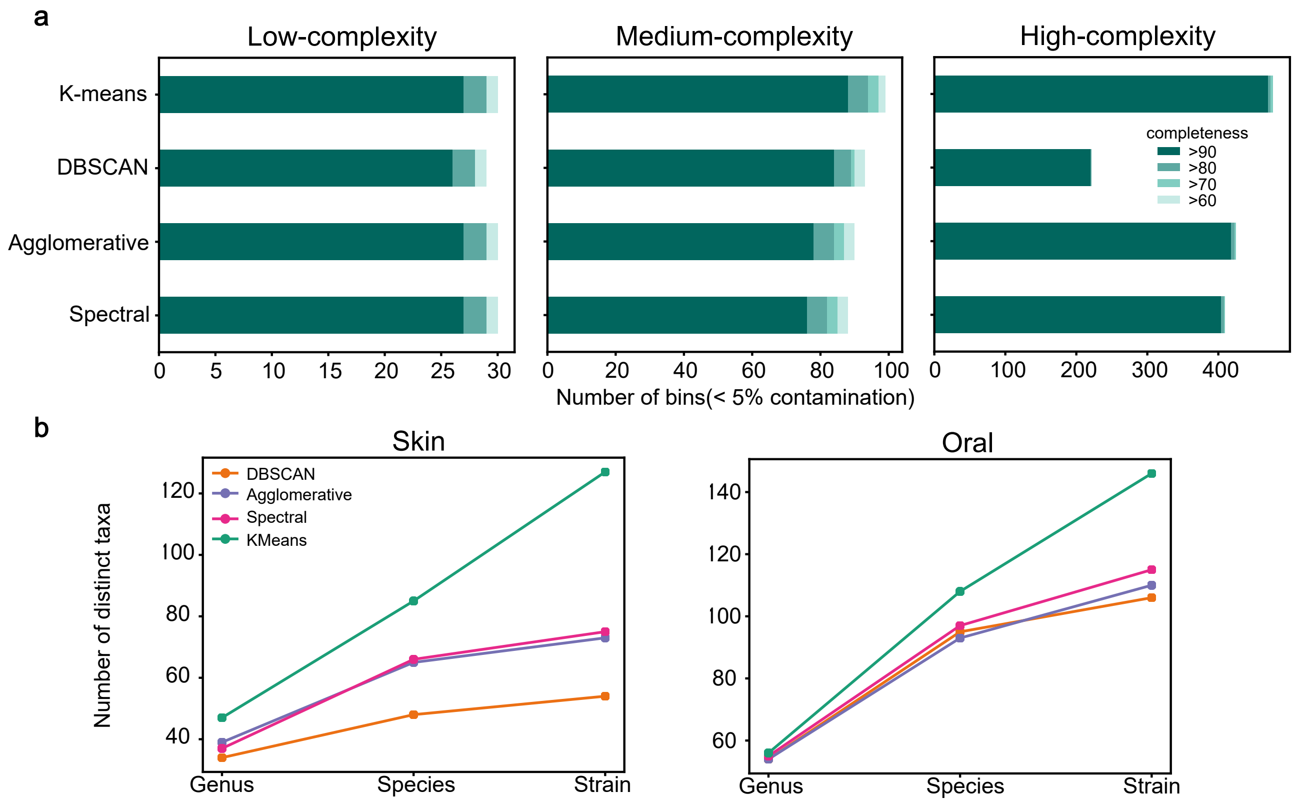

Supplementary Fig 24. Weighted *k*-means outperformed other clustering methods in the reclustering step. Shown are the binning results using different clustering methods for reclustering. **a,** The number of reconstructed genomes with varying completeness and contamination < 5% on CAMI I datasets and **b,** The numbers of high-quality distinct genera, species and strains on CAMI II datasets.

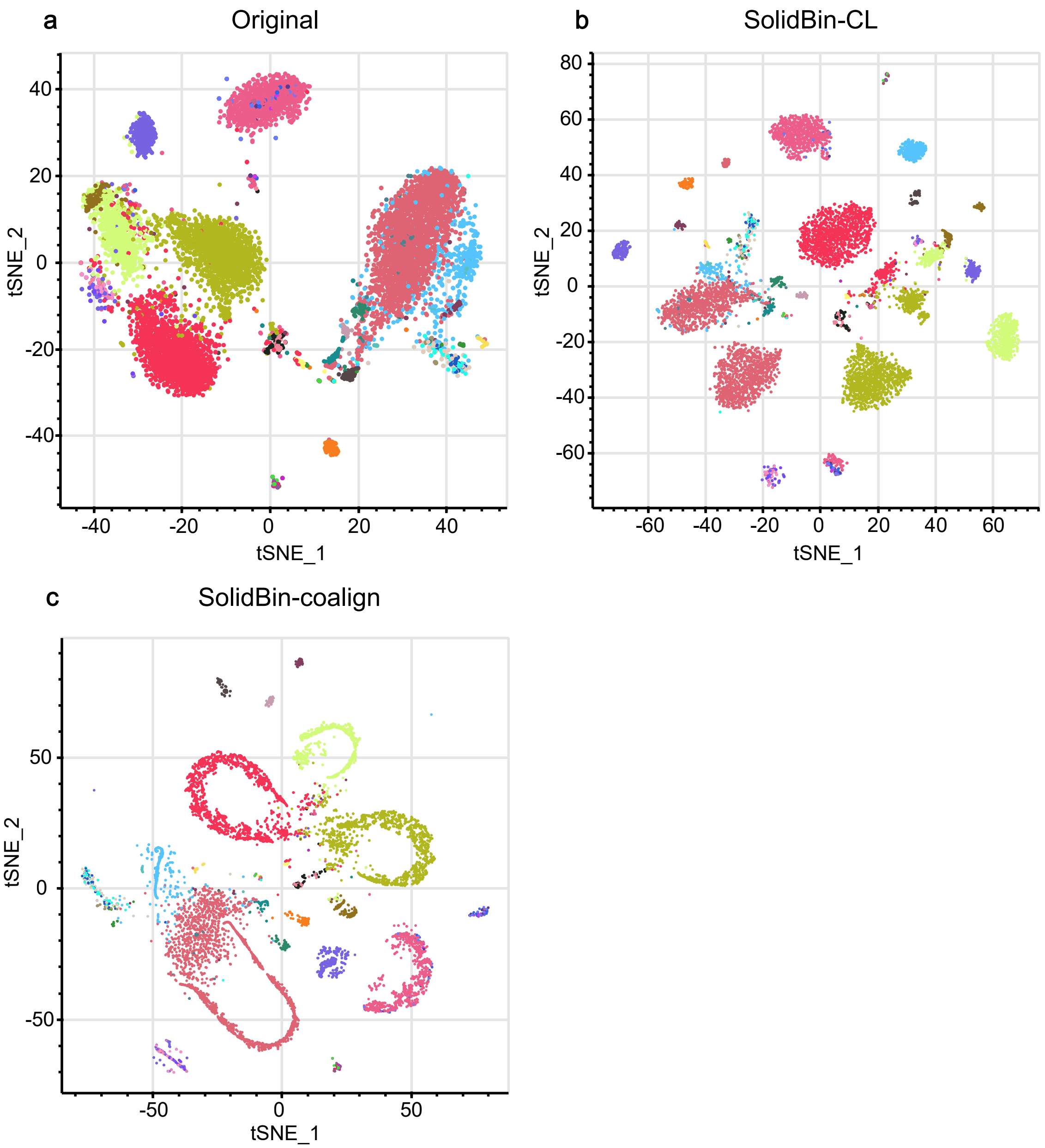

Supplementary Fig 25. Visualization of original features and embeddings from SolidBin in CAMI I low complexity dataset. Shown are **a,** the original features and embeddings from **b,** SolidBin-CL and **c,** SolidBin-coalign (two versions of SolidBin that used additional information from reference genomes) with t-SNE on the 40 genomes from CAMI I low complexity dataset. Fig. 2c shows the analogous result for SemiBin.

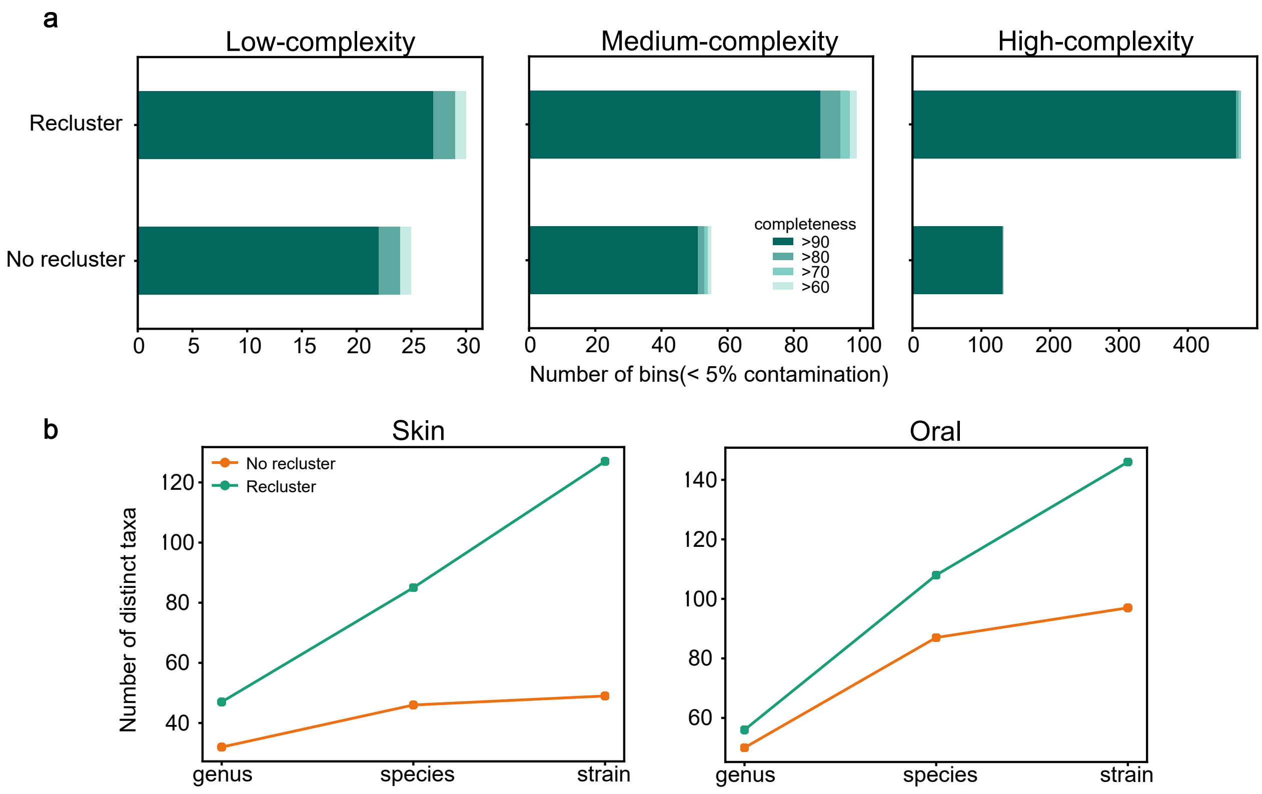

Supplementary Fig 26. Reclustering with weighted *k*-means can improve binning results on simulated datasets. We compared the results of binning with and without the reclustering step. Shown are **a,** the numbers of reconstructed genomes with varying completeness and contamination < 5% on CAMI I datasets and **b,** the numbers of high-quality distinct genera, species and strains on CAMI II datasets.

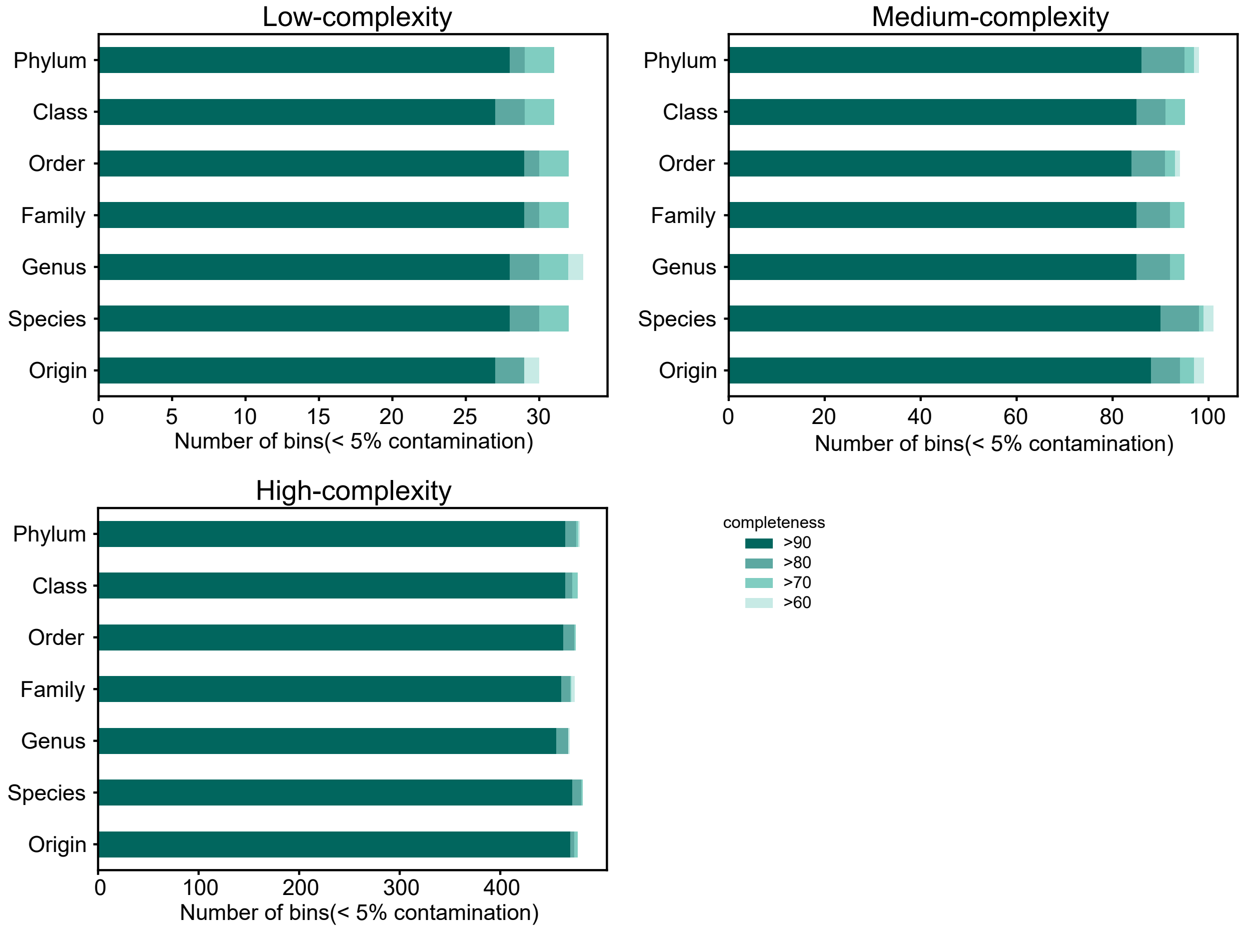

Supplementary Fig 27. SemiBin can be robust when removing related genomes from the GTDB. To evaluate if SemiBin's performance was overestimated in the CAMI datasets due to presence of related genomes in the annotation database, we removed genomes from GTDB that have the same taxonomic annotation with the genomes from CAMI I datasets at different taxonomic ranks: species, genus, family, order, class and phylum. Shown are the numbers of reconstructed genomes with varying completeness and contamination < 5% in CAMI I datasets. The results show that SemiBin does not depend on the presence of related genomes in the reference database. Even when excluding all genomes from the same phylum from the GTDB, we only observed a minor loss in the number of high-quality bins recovered.

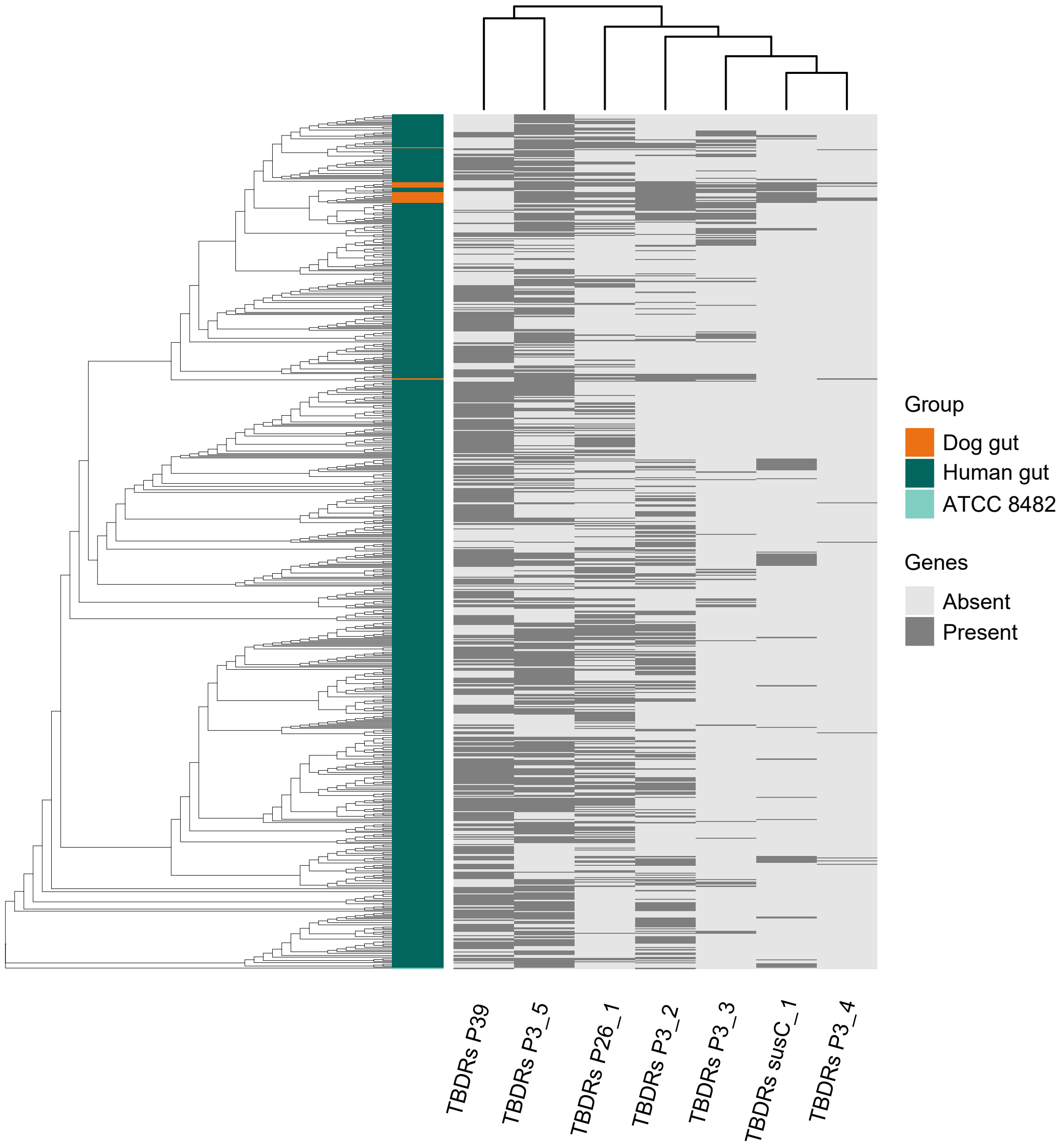

Supplementary Fig 28. The separation of *B. vulgatus* strains in human gut and dog gut could also be found with GMGCv1 as a external validation. We used 762 high-quality strains of *B. vulgatus* from the human gut in GMGCv1 as the external validation and a type strain *B. vulgatus* ATCC 8482. Shown are the maximum-likelihood phylogenetic trees based on core genes and the heatmap showing the presence or absence of the genes encoding TonB-dependent receptors (TBDR).

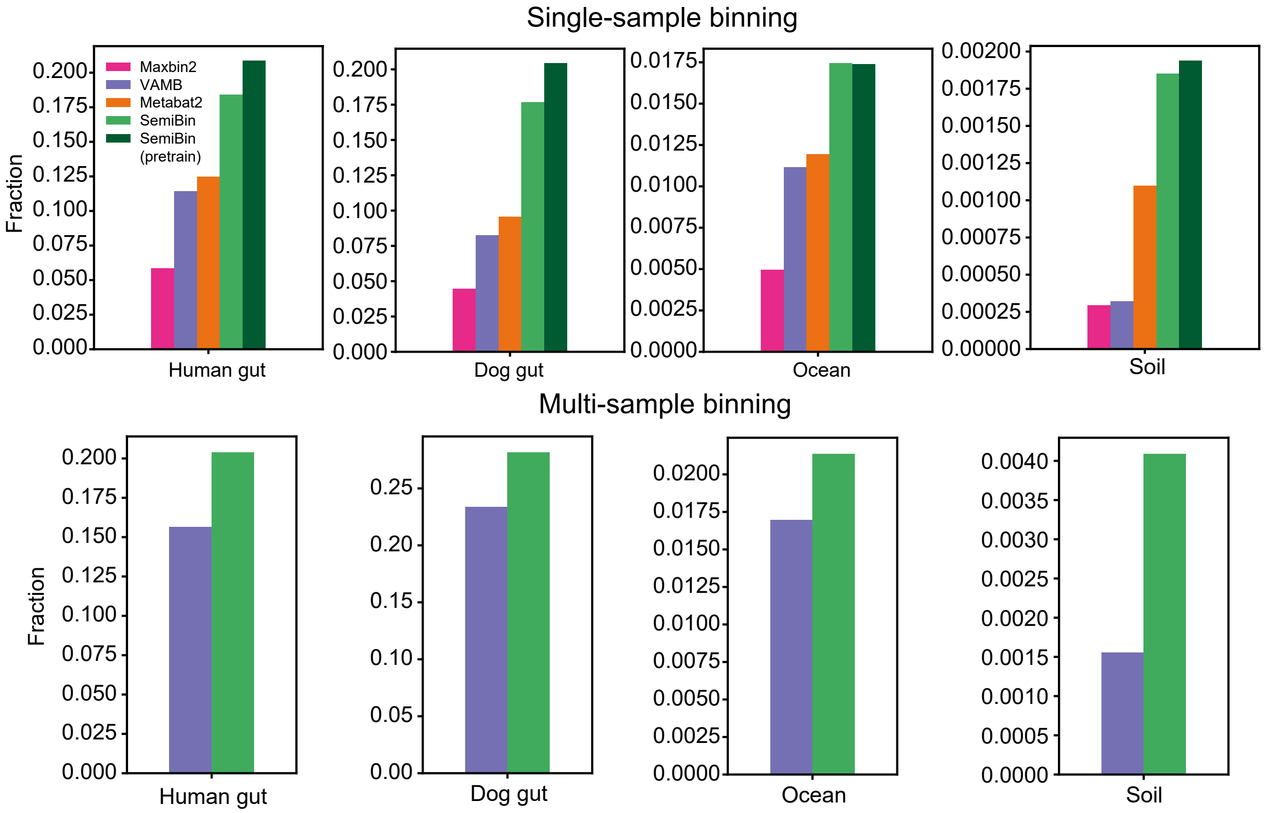

Supplementary Fig 29. SemiBin can bin a larger fraction of the assembly than other binners. Shown are the fractions of basepairs that can be binned as high-quality bins.

	Dataset	#Samples	#Genomes	Binning mode
CAMI I	Low complexity	1	40	Single-sample
	Medium complexity	2	132	Co-assembly
	High complexity	5	596	Co-assembly
CAMI II	Oral	10	799	Multi-sample
	Skin	10	610	Multi-sample
Real datasets	Dog gut	129	Unknown	Single/Multi-sample
	Human gut	82	Unknown	Single/Multi-sample
	Ocean	109	Unknown	Single/Multi-sample
	Soil	101	Unknown	Single/Multi-sample

Supplementary Table 1. Overview of the datasets used in the benchmarking.

Dataset	Nr. must-link	Must-link acc.	Nr. cannot-link	Cannot-link acc.	Nr. genomes	Nr. must-link covered genomes
Low	2,084,477	0.980	12,547,725	0.988	40	31
Medium	3,662,242	0.992	76,815,282	0.986	132	92
High	206,560	0.722	39,533,277	0.996	596	388
Skin_sample_1	2,346,244	0.250	58,684,337	0.994	90	66
Oral_sample_6	148,247	0.971	4,538,540	0.974	89	46
Oral_sample_7	302,528	0.285	11,557,221	0.995	179	109
Oral_sample_8	475,445	0.294	20,965,330	0.996	163	96
Skin_sample_13	430,244	0.170	3,224,103	0.982	140	35
Skin_sample_14	580,719	1.000	310,471	0.887	58	6
Skin_sample_15	2,286,104	0.125	14,402,383	0.988	72	39
Skin_sample_16	220,879	0.748	122,596	0.537	41	5
Skin_sample_17	806,282	0.537	28,461,743	0.995	89	53
Skin_sample_18	21,817	0.646	191,807	0.967	51	21
Skin_sample_19	3,432,141	0.334	271,153,441	0.998	241	154
Skin_sample_20	1,843,334	0.133	37,709,100	0.996	163	106
Skin_sample_28	109,276	0.999	213,438	0.895	25	3
Oral_sample_13	491,869	0.391	27,319,826	0.996	164	93
Oral_sample_14	696,582	0.548	39,416,022	0.997	141	88
Oral_sample_15	543,180	0.529	28,024,955	0.997	165	100
Oral_sample_16	570,645	0.573	25,082,966	0.994	320	85
Oral_sample_17	349,459	0.294	16,970,935	0.995	143	77
Oral_sample_18	570,501	0.678	33,019,031	0.997	150	82
Oral_sample_19	612,717	0.369	31,035,653	0.995	274	128

Supplementary Table 2. Accuracy of must-link and cannot-link constraints generated using MMseqs2 (GTDB reference) in simulated datasets. We used MMseqs2 to annotate contigs to the GTDB taxonomy. To test the quality of this annotation, we used the simulated datasets from CAMI I and CAMI II. Must-link acc: the accuracy of must-link constraints. Cannot-link acc: the accuracy of cannot-link constraints. Nr. genomes: number of genomes in the environment. Nr. must-link covered genomes: number of genomes that were covered by the accurate must-link constraints.

	#samples	Habitat(s)	Binning tool
Pasoli et al.[20]	9,428	Human associated	Metabat2
Coelho et al.[17]	13,174	Global microbiome	Metabat2
Almeida et al.[21]	11,850	Human gut	Metabat2
Nayfach et al.[22]	3,810	Human gut	DAS Tool(Maxbin2;Metabat2;CONCOCT)
Nayfach et al.[23]	> 10,000	Global microbiome	Metabat
Schulz et al.[24]	8,535	Global virome	Metabat2
Asnicar et al.[25]	1,203	Human gut	Metabat2

Supplementary Table 3. Binning tools used in recent large-scale metagenomic studies.

	NCBI		GTDB		
	Acc	#genomes	Acc	#genomes	#All genomes
Low	0.998	39	0.983	38	40
Medium	0.998	127	0.991	125	132
High	0.9997	567	0.9987	535	596

Supplementary Table 4. Accuracy of cannot-link constraints generated from NCBI and GTDB reference genomes on CAMI I datasets. Acc: accuracy of the cannot-link constraints. #genomes: number of genomes were covered by the accurate cannot-link constraints. #All genomes: number of genomes in the dataset.

		Single-sample			Single-sample(pretrain)			Multi-sample		
		Human gut	Dog gut	Ocean	Human gut	Dog gut	Ocean	Human gut	Dog gut	Ocean
Computing features	Time(min)	5	9	12	5	9	12	29	36	34
	Memory(MB)	923	736	1,675	923	736	1,675	807	784	798
Generating cannot-link	Time(min)	88	81	114	n/a	n/a	n/a	88	81	114
	Memory(MB)	39,070	37,904	46,091	n/a	n/a	n/a	39,070	37,904	46,091
Training(CPU)	Time(min)	181	209	222	n/a	n/a	n/a	184	210	222
	Memory(MB)	2,497	2,373	3,211	n/a	n/a	n/a	2,474	2,372	3,225
Training(GPU)	Time(min)	34	36	45	n/a	n/a	n/a	34	36	45
	Memory(MB)	4,487	4,355	5,222	n/a	n/a	n/a	4,491	4,364	5,233
Binning	Time(min)	2	2	3	2	2	3	2	2	2
	Memory(MB)	4,501	3,641	7,622	4,501	3,641	7,622	3,887	3,655	4,775

Supplementary Table 5. Running time and memory usage of SemiBin processing steps. For every environment, we randomly chose 10 samples to evaluate the running time and memory usage of SemiBin. Shown are the peak memory usage and average running time per sample, except computing features for multi-sample binning, where features are computed for all samples simultaneously. For benchmarking CPU time, we used an AWS g4ad.4xlarge machine with 1 CPU, 8 physical cores and 16 logical cores. For GPU machine, we used Tesla T4. GPU: Graphical Processing Unit, CPU: Central Processing Unit.

		Time(min)			Memory(MB)		
		Human gut	Dog gut	Ocean	Human gut	Dog gut	Ocean
Single-sample binning	Maxbin2	25.8	25.1	74.3	503	360	2,083
	VAMB(CPU)	7.0	8.9	14.1	321	322	1,014
	VAMB(GPU)	3.9	6.2	10.2	2,898	2,870	2,911
	Metabat2	1.1	2.0	4.9	4,892	2,735	4,892
	SemiBin(CPU)	276.0	300.8	350.9	39,070	37,904	46,091
	SemiBin(GPU)	128.5	127.6	173.1	39,070	37,904	46,091
	SemiBin(pretrain)	7.2	10.6	14.9	4,501	3,641	7,622
Multi-sample binning	VAMB(CPU)	10.3	10.6	17.9	1,085	1,291	918
	VAMB(GPU)	1.7	2.2	3.0	3,690	3,880	3,495
	SemiBin(CPU)	276.5	297.3	341.0	39,070	37,904	46,091
	SemiBin(GPU)	126.3	122.8	164.0	39,070	37,904	46,091

Supplementary Table 6. Running time and memory usage for the different binning tools. Average per sample time and peak memory usage (over 10 randomly chosen samples). For CPU timings, we used an AWS g4ad.4xlarge machine with 1 CPU, 8 physical cores and 16 logical cores. For GPU machine, we used a Tesla T4. GPU: Graphical Processing Unit, CPU: Central Processing Unit.

Dataset	Nr. contigs	Must-link fraction	Cannot-link fraction
Low	8,436	0.348	0.641
Medium	26,076	0.365	0.487
High	36,856	0.618	0.244
Skin_sample_1	23,903	0.516	0.466
Oral_sample_6	7,021	0.577	0.453
Oral_sample_7	10,764	0.464	0.473
Oral_sample_8	13,602	0.435	0.503
Skin_sample_13	5,963	0.477	0.475
Skin_sample_14	1,760	0.223	0.760
Skin_sample_15	12,990	0.620	0.455
Skin_sample_16	1,383	0.299	0.608
Skin_sample_17	20,363	0.744	0.378
Skin_sample_18	2,078	0.802	0.334
Skin_sample_19	51,126	0.526	0.465
Skin_sample_20	18,287	0.419	0.490
Skin_sample_28	1,261	0.393	0.655
Oral_sample_13	14,776	0.390	0.516
Oral_sample_14	17,349	0.366	0.523
Oral_sample_15	15,458	0.452	0.492
Oral_sample_16	15,325	0.448	0.471
Oral_sample_17	16,208	0.742	0.389
Oral_sample_18	19,300	0.737	0.430
Oral_sample_19	17,814	0.497	0.458

Supplementary Table 7. The fraction of contigs that have must-link and cannot-link constraints in the simulated datasets. Legend: Nr. contigs: the number of contigs used in the binning; Must-link fraction: the fraction of contigs that were broken up to obtain must-link constraints; Cannot-link fraction: the fraction of contigs that have cannot-link constraints

Supplementary References

1. Chen, T., Kornblith, S., Norouzi, M. & Hinton, G. A simple framework for contrastive learning of visual representations. In *International conference on machine learning*, 1597–1607 (PMLR, 2020).
2. DeMaere, M. Z. & Darling, A. E. bin3c: exploiting hi-c sequencing data to accurately resolve metagenome-assembled genomes. *Genome biology* **20**, 1–16 (2019).
3. Raghavan, U. N., Albert, R. & Kumara, S. Near linear time algorithm to detect community structures in large-scale networks. *Phys. review E* **76**, 036106 (2007).
4. Traag, V. A., Waltman, L. & Van Eck, N. J. From louvain to leiden: guaranteeing well-connected communities. *Sci. reports* **9**, 1–12 (2019).
5. Blondel, V. D., Guillaume, J.-L., Lambiotte, R. & Lefebvre, E. Fast unfolding of communities in large networks. *J. statistical mechanics: theory experiment* **2008**, P10008 (2008).
6. Csardi, G., Nepusz, T. *et al.* The igraph software package for complex network research. *InterJournal, complex systems* **1695**, 1–9 (2006).
7. Shi, J. & Malik, J. Normalized cuts and image segmentation. *IEEE Transactions on pattern analysis machine intelligence* **22**, 888–905 (2000).
8. Ester, M., Kriegel, H.-P., Sander, J., Xu, X. *et al.* A density-based algorithm for discovering clusters in large spatial databases with noise. In *kdd*, vol. 96, 226–231 (1996).
9. Maaten, L. v. d. & Hinton, G. Visualizing data using t-sne. *J. machine learning research* **9**, 2579–2605 (2008).
10. von Meijenfeldt, F. B., Arkhipova, K., Cambuy, D. D., Coutinho, F. H. & Dutilh, B. E. Robust taxonomic classification of uncharted microbial sequences and bins with cat and bat. *Genome biology* **20**, 1–14 (2019).
11. Steinegger, M. & Söding, J. Mmseqs2 enables sensitive protein sequence searching for the analysis of massive data sets. *Nat. biotechnology* **35**, 1026–1028 (2017).
12. Mirdita, M., Steinegger, M., Breitwieser, F., Soeding, J. & Levy Karin, E. Fast and sensitive taxonomic assignment to metagenomic contigs. *Bioinformatics* **37**, 3029–3031 (2021).
13. Chaumeil, P.-A., Mussig, A. J., Hugenholtz, P. & Parks, D. H. Gtdb-tk: a toolkit to classify genomes with the genome taxonomy database (2020).
14. Seemann, T. Prokka: rapid prokaryotic genome annotation. *Bioinformatics* **30**, 2068–2069 (2014).
15. Page, A. J. *et al.* Roary: rapid large-scale prokaryote pan genome analysis. *Bioinformatics* **31**, 3691–3693 (2015).
16. Nguyen, L.-T., Schmidt, H. A., Von Haeseler, A. & Minh, B. Q. Iq-tree: a fast and effective stochastic algorithm for estimating maximum-likelihood phylogenies. *Mol. biology evolution* **32**, 268–274 (2015).

17. Coelho, L. P. *et al.* Towards the biogeography of prokaryotic genes. *Nature* 1–5 (2022).

18. Parks, D. H., Imelfort, M., Skennerton, C. T., Hugenholtz, P. & Tyson, G. W. Checkm: assessing the quality of microbial genomes recovered from isolates, single cells, and metagenomes. *Genome research* **25**, 1043–1055 (2015).

19. Orakov, A. *et al.* Gunc: detection of chimerism and contamination in prokaryotic genomes. *Genome biology* **22**, 1–19 (2021).

20. Pasolli, E. *et al.* Extensive unexplored human microbiome diversity revealed by over 150,000 genomes from metagenomes spanning age, geography, and lifestyle. *Cell* **176**, 649–662 (2019).

21. Almeida, A. *et al.* A new genomic blueprint of the human gut microbiota. *Nature* **568**, 499–504 (2019).

22. Nayfach, S., Shi, Z. J., Seshadri, R., Pollard, K. S. & Kyrpides, N. C. New insights from uncultivated genomes of the global human gut microbiome. *Nature* **568**, 505–510 (2019).

23. Nayfach, S. *et al.* A genomic catalog of earths microbiomes. *Nat. biotechnology* 1–11 (2020).

24. Schulz, F. *et al.* Giant virus diversity and host interactions through global metagenomics. *Nature* **578**, 432–436 (2020).

25. Asnicar, F. *et al.* Microbiome connections with host metabolism and habitual diet from 1,098 deeply phenotyped individuals. *Nat. Medicine* **27**, 321–332 (2021).
