# Supplementary material for: A deep siamese neural network improves metagenome-assembled genomes in microbiome datasets across different environments
Source: Nat Commun. 2022 Apr 28;13:2326. doi: 10.1038/s41467-022-29843-y (PMC9051138; doi:10.1038/s41467-022-29843-y)
Supplement: Supplementary file 3 — Description of Additional Supplementary Files [file 41467_2022_29843_MOESM3_ESM.docx]

File Name: Supplementary Data 1

Description: Significantly different Tonb and btub genes found in Bacteroides vulgatus strains from human gut and dog gut datasets.

File Name: Supplementary Data 2

Description: Soil samples used in the benchmarking.
