# Supplementary material for: A deep siamese neural network improves metagenome-assembled genomes in microbiome datasets across different environments
Source: Nat Commun. 2022 Apr 28;13:2326. doi: 10.1038/s41467-022-29843-y (PMC9051138; doi:10.1038/s41467-022-29843-y)
Supplement: Supplementary file 6 — Reporting Summary [file 41467_2022_29843_MOESM6_ESM.pdf]

nature portfolio

Corresponding author(s): Xing-Ming Zhao; Luis Pedro Coelho

Last updated by author(s): Mar 28, 2022

Reporting Summary

Nature Portfolio wishes to improve the reproducibility of the work that we publish. This form provides structure for consistency and transparency in reporting. For further information on Nature Portfolio policies, see our Editorial Policies and the Editorial Policy Checklist.

Statistics

For all statistical analyses, confirm that the following items are present in the figure legend, table legend, main text, or Methods section.

n/a	Confirmed	
☐	☒	The exact sample size (n) for each experimental group/condition, given as a discrete number and unit of measurement
☐	☒	A statement on whether measurements were taken from distinct samples or whether the same sample was measured repeatedly
☐	☒	The statistical test(s) used AND whether they are one- or two-sided<br>*Only common tests should be described solely by name; describe more complex techniques in the Methods section.*
☐	☒	A description of all covariates tested
☒	☐	A description of any assumptions or corrections, such as tests of normality and adjustment for multiple comparisons
☐	☒	A full description of the statistical parameters including central tendency (e.g. means) or other basic estimates (e.g. regression coefficient) AND variation (e.g. standard deviation) or associated estimates of uncertainty (e.g. confidence intervals)
☐	☒	For null hypothesis testing, the test statistic (e.g. F, t, r) with confidence intervals, effect sizes, degrees of freedom and P value noted<br>*Give P values as exact values whenever suitable.*
☒	☐	For Bayesian analysis, information on the choice of priors and Markov chain Monte Carlo settings
☒	☐	For hierarchical and complex designs, identification of the appropriate level for tests and full reporting of outcomes
☒	☐	Estimates of effect sizes (e.g. Cohen's d, Pearson's r), indicating how they were calculated

Our web collection on statistics for biologists contains articles on many of the points above.

Software and code

Policy information about availability of computer code

Data collection	No software was used.
Data analysis	For the development of SemiBin, we used Python v.3.7, Pytorch v.1.8, python-igraph v.0.9.7, MMseq2 v.13.45111, FragGeneScan v.1.30, BEDTools v.2.29.1, HMMER v.3.1b1. You can download SemiBin from https://github.com/BigDataBiology/SemiBin/.<br><br>For the analysis of the benchmarking results, we used fastANI v.1.32, Maxbin2 v.2.2.6, Metabat2 v.2, VAMB.v.3.0.2, COCACOLA, SolidBin.v.1.3, AMBER v.2.0.1, CheckM v.1.1.3, Mash v.2.2, GTDB-Tk v.1.4.1, Prokka v.1.14.5, Roary v.3.13.0, Scoary v.1.6.16, IQTREE v1.6.9, NGLess v.1.0.1, Megahit v.1.2.4, Bowtie2 v.2.4.1. All the codes for the benchmark, visualization and analysis can be found here: https://github.com/BigDataBiology/SemiBin_benchmark.

For manuscripts utilizing custom algorithms or software that are central to the research but not yet described in published literature, software must be made available to editors and reviewers. We strongly encourage code deposition in a community repository (e.g. GitHub). See the Nature Portfolio guidelines for submitting code & software for further information.

Data

Policy information about availability of data

All manuscripts must include a data availability statement. This statement should provide the following information, where applicable:

- Accession codes, unique identifiers, or web links for publicly available datasets
- A description of any restrictions on data availability
- For clinical datasets or third party data, please ensure that the statement adheres to our policy

The sequence data used in the study are publicly available in the ENA with study accessions PRJEB27928, PRJEB20308, PRJEB1787, PRJEB1788, PRJEB4352, PRJEB4419, PRJNA504891, PRJNA290729, PRJEB4391, PRJEB6997, PRJEB7759, PRJEB11755, PRJNA271013 and PRJNA300541. The study accessions of the soil dataset see Supplementary Data 2. The simulated CAMI I(low, medium and high complexity) and CAMI II datasets(skin and oral cavity from Toy Human Microbiome Project Dataset) can be downloaded from https://data.cami-challenge.org/participate. The MAGs that generated from real metagenomes in the benchmarking can be obtained from Zenodo: DOI: 10.5281/zenodo.5181237 (human gut microbiome MAGs), DOI: 10.5281/zenodo.5181385 (dog gut microbiome MAGs), DOI: 10.5281/zenodo.5181391 (marine microbiome MAGs) and DOI: 10.5281/zenodo.5861178 (soil microbiome MAGs). All intermediate results of benchmarking can be found on Github at https://github.com/BigDataBiology/SemiBin_benchmark. Source data are provided with this paper.

Field-specific reporting

Please select the one below that is the best fit for your research. If you are not sure, read the appropriate sections before making your selection.

- [x] Life sciences
- [ ] Behavioural & social sciences
- [ ] Ecological, evolutionary & environmental sciences

For a reference copy of the document with all sections, see nature.com/documents/nr-reporting-summary-flat.pdf

Life sciences study design

All studies must disclose on these points even when the disclosure is negative.

Sample size	No calculation of sample sizes were made. We used the available public datasets: CAMI I low (n = 1), CAMI I medium (n = 2), CAMI I high (n = 5), CAMI II skin (n = 10), CAMI II oral (n = 10), German human gut (n = 82), Dog gut (n = 129), Marine (n = 109), soil (n = 101), German human gut (n = 92), African human gut (n = 50), cat gut (n = 30), human oral (n = 30), mouse gut (n = 30), pig gut (n = 30), built environment (n = 30) and wastewater (n = 17). For the simulated datasets, we used all the samples in the datasets. For the real datasets benchmarking, we used samples from the corresponding project to evaluate the performance. We used 82, 129, 109, 101 samples, which is enough for the benchmarking. For the training and testing of six extra environments, we used 10 samples to test the model and the extra samples to train the model.
Data exclusions	Data from four habitats that were available in GMGCv1 was not used as there were not enough samples of sufficient sequencing depth. When multiple samples from the same individual were present, only one was used.
Replication	As described above ("Sample size"), multiple samples from the same habitat were used and three human gut datasets were used.
Randomization	Several metrics were evaluated on a randomly selected subset of samples.<br><br>When splitting into training and testing, samples with the lowest accession numbers were chosen as training and the rest as testing. While not random, across a variety of different datasets, we consider this an unbiased, yet reproducible, procedure.
Blinding	Investigators were not blind to the datasets.

Reporting for specific materials, systems and methods

We require information from authors about some types of materials, experimental systems and methods used in many studies. Here, indicate whether each material, system or method listed is relevant to your study. If you are not sure if a list item applies to your research, read the appropriate section before selecting a response.

Materials & experimental systems

n/a	Involved in the study	
[x]	[ ]	Antibodies
[x]	[ ]	Eukaryotic cell lines
[x]	[ ]	Palaeontology and archaeology
[x]	[ ]	Animals and other organisms
[x]	[ ]	Human research participants
[x]	[ ]	Clinical data
[x]	[ ]	Dual use research of concern

Methods

n/a	Involved in the study	
[x]	[ ]	ChIP-seq
[x]	[ ]	Flow cytometry
[x]	[ ]	MRI-based neuroimaging
